# The KIF6‐RBP Complex Orchestrates mRNA Transport Required for Sperm Flagellar Assembly

**DOI:** 10.1002/advs.76263

**Published:** 2026-06-25

**Authors:** Chunbo Xie, Sibing Yi, Xinle Lin, Shen Zhang, Weili Wang, Shimin Yuan, Lanlan Meng, Yong Li, Chen Tan, Chunjia Wei, Yanyan Yu, Yaoqiong Liang, Huan Zhang, Liang Hu, Guangxiu Lu, Wenbin He, Qianjun Zhang, Juan Du, Ge Lin, Chaofeng Tu, Yue‐Qiu Tan

**Affiliations:** ^1^ Institute of Reproduction and Stem Cell Engineering Xiangya School of Basic Medical Sciences Central South University Changsha China; ^2^ Clinical Research Center for Reproduction and Genetics in Hunan Province Reproductive & Genetic Hospital of CITIC‐Xiangya Changsha China; ^3^ NHC Key Laboratory of Human Stem Cell and Reproductive Engineering Xiangya School of Basic Medical Sciences Central South University Changsha China; ^4^ College of Life Sciences Hunan Normal University Changsha China

**Keywords:** asthenozoospermia, flagellar assembly, KIF6, mRNA transport, RNA‐binding protein

## Abstract

The precise assembly of the sperm flagellum is essential for male fertility and has long been ascribed to kinesin‐2–driven intraflagellar transport (IFT) of protein cargoes. However, during late spermiogenesis, when transcription activity is largely silenced, how the spatiotemporally regulated delivery of flagellar components remains poorly understood. Here, we systematically screened kinesin genes in asthenozoospermic patients and identified two homozygous deleterious *KIF6* variants in unrelated men characterized by complete sperm immotility. Mouse models carrying the corresponding mutations recapitulated the human infertility phenotypes. Multi‐omics analyses revealed that most testicular mRNAs remained largely unchanged in *Kif6* ^M1/M1^ mice, whereas proteins involved in axonemal organization and energy metabolism were markedly reduced. Mechanistically, KIF6 interacts with the RNA‐binding proteins (RBPs) FMRP and FXR1 to assemble mRNP transport complexes that ferry transcripts encoding flagellar structural proteins (e.g., DNALI1) and metabolic enzymes (e.g., HK1). Impaired KIF6 function compromises mRNP trafficking to the developing flagellum, reducing flagellar transcript levels and ultimately causing decreased protein abundance and defective flagellar function. Collectively, we identify KIF6 as a key regulator of mRNA transport during spermiogenesis. It interacts with RBPs through a novel IFT‐like pathway to deliver mRNAs essential for flagellar biogenesis, redefining the traditional protein‐centric IFT paradigm.

## Graphical abstract

Two homozygous deleterious *KIF6* variants are identified in unrelated men with impaired sperm motility. Mouse models and multi‐omics analyses reveal that KIF6 cooperates with the RNA‐binding proteins FMRP and FXR1 to deliver mRNAs essential for sperm flagellar assembly, linking disrupted mRNA transport to reduced abundance of key structural and metabolic protein and male infertility.

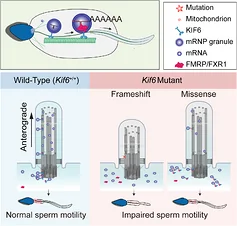

## 1 Introduction

The sperm flagellum is a specialized microtubule‐based organelle essential for male fertility and sexual reproduction [1]. Its conserved “9+2” axonemal architecture spans eukaryotic evolution, from lower plants such as mosses and ferns to animals including *Drosophila* and humans, underscoring its fundamental role in cell motility across diverse biological contexts, ranging from protozoan locomotion to mammalian fertilization [2, 3, 4, 5, 6]. In humans, defects in flagellar structure and function directly lead to asthenozoospermia and male infertility [7, 8, 9].

The canonical intraflagellar transport (IFT) pathway, which is evolutionarily conserved from *Chlamydomonas* to humans [10], has long been considered the linchpin of flagellar assembly. In this pathway, kinesin‐2 family motors (including KIF3A, KIF3B, KIF3C and KIF17) mediate the anterograde transport of cytoplasmically synthesized structural and membrane proteins along axonemal microtubules toward the distal tip of the flagellum [11, 12, 13]. Notably, additional kinesin family members are also expressed in the testis; however, whether these kinesins participate in and support flagellar assembly through non‐canonical transport mechanisms remains unknown.

The kinesin superfamily, highly conserved microtubule‐dependent motors, includes 14 families (from kinesin‐1 to kinesin‐14) and 45 kinesins [14]. Their functions extend beyond canonical IFT‐mediated protein transport to include microtubule‐dependent mRNA localization [15, 16, 17], suggesting the existence of non‐canonical transport processes. For example, in *Drosophila* oocytes, kinesin‐1 delivers *oskar* mRNA to the posterior pole, thereby establishing embryonic polarity [18]. In mammalian neurons, kinesins associate with RNA‐binding proteins (RBPs), such as FMRP and APC, to promote the directional transport of mRNA granules to synapses [19, 20, 21]. In male germ cells, KIF17b transports CREM‐regulated mRNAs, including those involved in spermatid differentiation, during spermatogenesis [22].

Moreover, motile cilia share striking structural and developmental homology with sperm flagella [23, 24], offering a valuable framework to dissect conserved mechanisms of organelle assembly. Accumulating evidence indicates that motile cilia harbor translational machinery capable of supporting local protein synthesis [25, 26, 27, 28]. For instance, FMRP mediates the delivery of specific mRNAs to mouse ependymal cilia and regulates their local translation [25]. During spermiogenesis, global transcriptional activity is largely quiescent. Spermiogenic mRNAs, including those encoding flagellar assembly components, are transcribed earlier and stored as translationally repressed messenger ribonucleoprotein (mRNP) granules [29]. In late spermiogenesis, a subset of these transcripts is subsequently translated in the sperm head [13]. Notably, paralleling observations in motile cilia, biochemical fractionation, electron microscopy‐based in situ hybridization, proteomic analyses, and metabolic labeling studies have detected nuclear‐encoded mRNAs, ribosomal subunits, and nascent peptides within sperm flagella [30, 31, 32, 33]. Together, these findings provide molecular and spatial evidence consistent with an intrinsic translational potential within the flagellum. However, how flagellum‐destined mRNAs are selectively transported to this compartment, and whether kinesins participate in and support this process, remain poorly understood.

To address this critical gap, we systematically prioritized candidate kinesins in humans through integrated expression and genetic screening and identified KIF6 as the top candidate for comprehensive functional investigation using CRISPR‐engineered mouse models and multi‐omics approaches. Men harboring homozygous deleterious *KIF6* variants presented with asthenozoospermia characterized by complete sperm immotility, a phenotype that is reflected in allele‐specific reproductive defects in *Kif6*‐mutant mice. Mechanistically, KIF6 forms a functional complex with the RBPs FMRP and FXR1 to bind and transport mRNAs encoding flagellar structural and metabolic components to the assembling flagellum. These findings uncover a non‐canonical, kinesin–RBP‐dependent, IFT‐like transport mechanism that mediates directed mRNA transport during spermiogenesis, expanding the protein‐centric framework of flagellar assembly and establishing KIF6 deficiency as a novel genetic etiology of human male infertility.

## 2 Results

### 2.1 Homozygous Deleterious *KIF6* Variants Are Strongly Associated With Asthenozoospermia

To identify non‐canonical kinesin regulators of sperm flagellar biogenesis, we specifically focused on N‐kinesins—plus‐end‐directed motors that mediate intracellular cargo transport and contribute to ciliary structure maintenance—while excluding established core intraflagellar transport (IFT) components (KIF3A, KIF3B, KIF3C and KIF17) [12]. We integrated complementary expression‐based and genetic screening strategies (Figure S1). First, transcriptomic data from the Human Protein Atlas were interrogated to identify N‐kinesin genes enriched in the testis, yielding six candidates: *KIF6*, *KIF20A*, *KIF9*, *KIF18A*, *KIF27* and *KIF15*. Protein expression profiling further revealed that KIF6 exhibits tissue‐restricted distribution, predominantly in the testis and other motile ciliated tissues, including bronchus, nasopharynx and fallopian tube, rather than broad expression across somatic tissues. This pattern suggests a close association with motile ciliary structures. Second, whole‐exome sequencing was performed in 314 men with asthenozoospermia and 1500 fertile controls. Following stringent variant filtering, rare homozygous variants were identified in four N‐kinesin genes—*KIF6*, *KIF13B*, *KIF20B*, and *KIF23* (Table S1). Notably, KIF6 was the only gene independently prioritized by both expression‐based and genetic screening approaches. Together with the evolutionary conservation of KIF6 across flagellated species (Figure S2A) and previous evidence demonstrating its directional motility along ciliary microtubules [34], these findings further support a critical role for KIF6 in sperm flagellar assembly.

**FIGURE 1:**
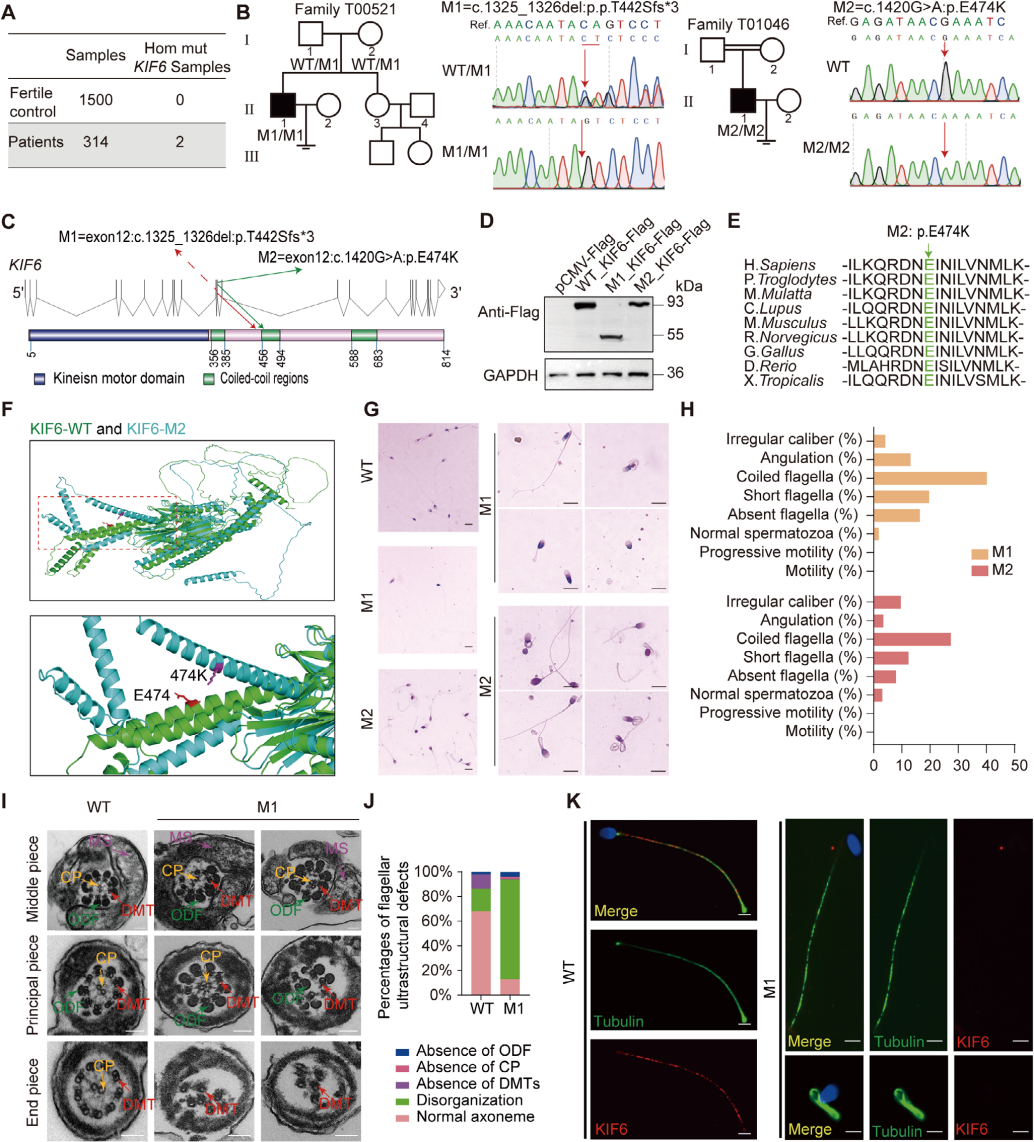
Identification of homozygous deleterious *KIF6* variants in infertile men with asthenozoospermia. (A) Overview of the cohort analyzed in this study, including 314 individuals diagnosed with asthenozoospermia and 1500 fertile controls. (B) Whole‐exome sequencing identified two homozygous *KIF6* variants in probands from two unrelated families: proband F1‐II‐2 carrying a frameshift variant (c.1325_1326del, p.T442Sfs*3) and proband F2‐II‐1 carrying a missense variant (c.1420G >A, p.E474K). Sanger sequencing confirmed both variants, with red arrows indicating the mutated nucleotides. (C) Schematic representation of the *KIF6* transcript and corresponding protein domain organization, indicating the positions of the p.T442Sfs*3 (red) and p.E474K (green) variants. (D) Representative Western blotting of KIF6 in HEK293T cells expressing WT or patient‐derived mutant constructs. GAPDH served as a loading control. (E) Cross‐species alignment of KIF6 sequences showing conservation of the E474K residue among mammals (green arrow). (F) Predicted structural models of WT KIF6 (AF‐Q6ZMV9‐F1‐v6) from the AlphaFold Protein Structure Database and the p.E474K variant generated using AlphaFold3. (G) Representative Papanicolaou staining of spermatozoa from a fertile donor from sperm bank with normal *KIF6* genotype (WT) and individuals carrying homozygous *KIF6* variants. Scale bars, 10 µm. (H) Quantification of sperm motility and flagellar categories in individuals carrying *KIF6* variants (n = 200 spermatozoa per individual). (I) Representative TEM cross‐sections of sperm flagella from the patient F1‐II‐2 carrying *KIF6* M1 homozygous variants. CP, central pair microtubules (yellow arrow); DMT, doublet microtubule (red arrow); MS, mitochondrial sheath (purple arrow); ODF, outer dense fiber (green arrow). Scale bars, 100 nm. (J) Quantification of different categories of flagellar ultrastructural defects. Total cross‐section numbers for quantification in the control individual and men harboring homozygous deleterious *KIF6* variant (subject F1‐II‐2) were 106 and 138, respectively. (K) Representative immunofluorescence staining of KIF6 in spermatozoa from a sperm donor with normal *KIF6* genotype (WT) and patient F1‐II‐2 carrying *KIF6* M1 homozygous variants. Scale bars, 5 µm. Abbreviations: M, mutation; WT, wild‐type.

Two independent homozygous *KIF6* variants were identified in unrelated individuals with complete sperm immotility: a frameshift variant (c.1325_1326del, p.T442Sfs*3) and a missense variant (c.1420G >A, p.E474K) (Figure 1A,B and Table S2). Both variants were extremely rare or absent in population databases (Table S3), and were not detected in our cohort of fertile controls, consistent with an autosomal recessive mode of inheritance. To assess the potential molecular consequences of these variants, we examined their predicted effects on KIF6 protein integrity and functional domains. *KIF6* encodes an 814–amino acid protein comprising an N‐terminal motor domain followed by three C‐terminal coiled‐coil domains. The p.T442Sfs*3 variant lies between the first and second coiled‐coil domains and is predicted to produce a truncated protein (Figure 1C). Consistent with this prediction, Western blot analysis of transfected HEK293T cells confirmed the expression of the expected shorter product (Figure 1D). The p.E474K variant alters a highly conserved residue within the second coiled‐coil domain (Figure 1C,E), and is predicted to disrupt local folding (Figure 1F).

To establish the genotype–phenotype correlation, we next characterized the sperm phenotypes of the affected individuals. Papanicolaou staining revealed pronounced flagellar abnormalities, including shortened, coiled, and irregularly thickened tails (Figure 1G,H and Table S2). Transmission electron microscopy (TEM) of spermatozoa from F1‐II‐2 further demonstrated severe ultrastructural defects, including disorganization of the mitochondrial sheath, disruption of peripheral dense fibers, and abnormalities in the canonical “9+2” axonemal architecture, in contrast to the well‐organized structures observed in fertile controls (Figure 1I,J). Immunofluorescence analysis showed that KIF6 localized along the entire flagellum in control spermatozoa, whereas its signal was nearly undetectable in spermatozoa from F1‐II‐2 (Figure 1K).

Finally, to assess whether infertility associated with KIF6 deficiency could be overcome by assisted reproductive technology, we analyzed intracytoplasmic sperm injection (ICSI) outcomes for F1‐II‐2. One ICSI cycle yielded a fertilization rate of 67% (8/12 metaphase II oocytes), with all embryos cleaving and four reaching the 8‐cell stage. Embryo transfer resulted in a live birth (Table S4), indicating that fertilization competence was preserved despite severe flagellar defects.

Together, these findings establish a strong genetic, molecular, and phenotypic association between homozygous deleterious *KIF6* variants and severe asthenozoospermia in humans, identifying KIF6 as a critical determinant of sperm flagellar motility.

### 2.2 *Kif6* Mutant Mice Recapitulate Key Features of Human *KIF6*‐Associated Asthenozoospermia and Reduced Male Fertility

KIF6 orthologs are evolutionarily conserved across diverse flagellated species (Figure S2A), highlighting the preservation of its fundamental functional architecture. Consistent with its putative role in spermatogenesis, *Kif6* is highly expressed in mouse testes (Figure S2B). Developmental profiling further showed that *Kif6* transcripts peak around postnatal day 14 and remain abundant throughout adulthood (Figure S2C). Immunofluorescence staining of spermatogenic cells further demonstrated that KIF6 is enriched in elongating spermatids (Figure S2D), coinciding with the developmental stage when flagellar assembly occurs.

**FIGURE 2:**
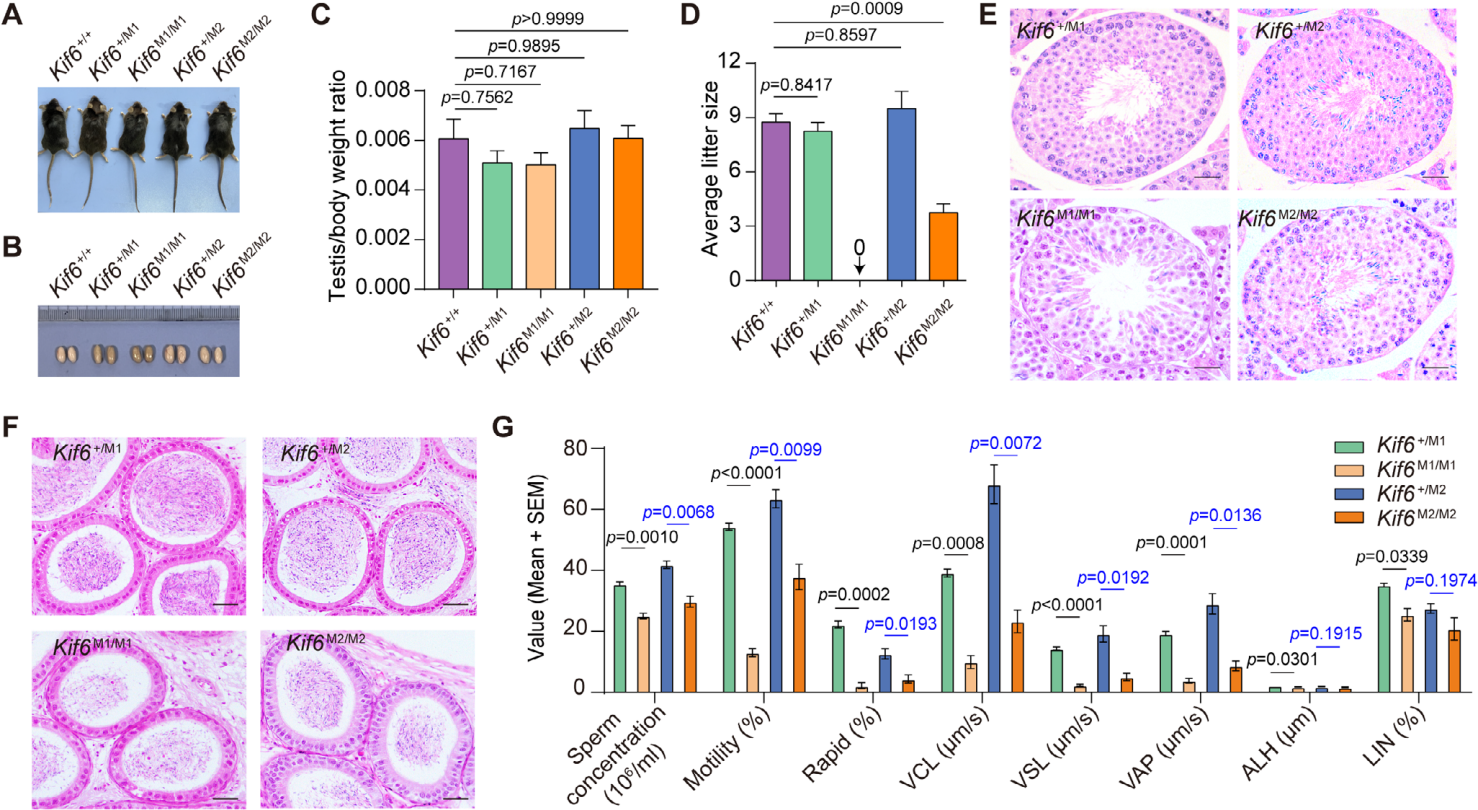
*Kif6* mutant mice (*Kif6* ^M1/M1^ and *Kif6* ^M2/M2^) recapitulate key features of human *KIF6*‐associated asthenozoospermia and reduced male fertility. (A) Gross morphology of adult *Kif6* mutant mice (*Kif6* ^M1/M1^ and *Kif6* ^M2/M2^), WT (*Kif6* ^+/+^) littermates and heterozygous mice (*Kif6* ^+/M1^ and *Kif6* ^+/M2^). (B) Representative images of testes from adult *Kif6* mutant mice, heterozygous mice, and WT littermates. (C) Testis‐to‐body weight ratios in *Kif6* mutant, heterozygous, and WT mice. Data are mean ± SEM (n = 3 mice per genotype). (D) Fertility assessment of *Kif6* mutant males following mating with proven fertile WT females. Litter size per mating is shown as mean ± SEM (n = 4 mice per genotype). (E) Representative H&E staining of testicular sections from heterozygous and *Kif6* mutant mice. Scale bars, 50 µm. (F) Representative H&E staining of caudal epididymal sections from heterozygous and *Kif6* mutant mice. Scale bars, 50 µm. (G) CASA of caudal sperm from *Kif6* mutant and control mice. Quantified parameters include average path velocity (VAP), curvilinear velocity (VCL), amplitude of lateral head displacement (ALH), straight‐line velocity (VSL), and linearity (LIN). Data are mean ± SEM (*n* = 3 mice per genotype). *P* values were calculated by one‐way ANOVA with Dunnett's T3 correction for multiple comparisons (C, D), or by unpaired two‐tailed Student's *t*‐test with Welch's correction (G).

To investigate the functional role of KIF6 in human spermatogenesis, we generated two CRISPR‐Cas9 mouse models, *Kif6* ^M1/M1^ (p.I486Ffs*10) and *Kif6* ^M2/M2^ (p.E473K), mimicking the human frameshift and missense variants, respectively (Figure S3A,B). At the molecular level, as expected, the frameshift variant in *Kif6* ^M1/M1^ mice led to the absence of detectable KIF6 protein (Figure S3C–F), and the missense variant in *Kif6* ^M2/M2^ mice produced a full‐length KIF6 protein (Figure S3E–G). Although the M1 cDNA produced a truncated KIF6 product in 293K cells, mutant *Kif6* transcripts were detectable in *Kif6* ^M1/M1^ mice, whereas Western blotting failed to detect the corresponding truncated protein, suggesting that the endogenous mutant transcript/protein may be limited by inefficient translation and/or reduced protein stability. At the organ level, testis size and weight were comparable among *Kif6* ^M1/M1^, *Kif6* ^M2/M2^, heterozygous, and wild‐type (WT, *Kif6* ^+/+^) males (Figure 2A–C), indicating that gross testicular development is preserved. Functional assessment revealed pronounced reproductive defects in both mutants, with *Kif6* ^M1/M1^ males being completely infertile and *Kif6* ^M2/M2^ males exhibiting significantly reduced fertility (Figure 2D). Histopathological evaluation of testicular and epididymal sections by hematoxylin and eosin (H&E) staining showed a marked reduction in sperm count in *Kif6* ^M1/M1^ males and a slight reduction in *Kif6* ^M2/M2^ males (Figure 2E,F), likely attributable to increased germ cell apoptosis (Figure S3H). PNA and acetylated tubulin co‐staining was further performed to examine the process of spermatid differentiation in testicular sections. Compared with *Kif6* ^+/+^ testes, in which abundant acetylated tubulin‐positive flagellar signals were observed in the seminiferous tubule lumens, *Kif6* ^M1/M1^ testes contained only sparse and short flagellar structures. *Kif6* ^M2/M2^ testes also showed fewer acetylated tubulin‐positive flagellar signals (Figure S4). Computer‐assisted sperm analysis (CASA) further demonstrated significantly reduced sperm concentration and impaired motility in *Kif6* mutant males relative to WT mice (Figure 2G).

Additionally, we occasionally observed hydrocephalus in *Kif6* ^M1/M1^ mice. Approximately 30% of *Kif6* ^M1/M1^ mice (17/57) developed hydrocephalus (Figure S5A,B), whereas no hydrocephalus was observed in *Kif6* ^M2/M2^ mice during the same observation period. Gross brain morphology and H&E staining confirmed ventricular enlargement in the affected *Kif6* ^M1/M1^ mice (Figure S5C). Given that KIF6 is also expressed in other motile ciliated tissues, we further examined respiratory motile cilia in hydrocephalic *Kif6* ^M1/M1^ mice to determine whether this phenotype was accompanied by defects in other motile ciliated tissues. TEM analysis showed no obvious axonemal ultrastructure abnormalities of respiratory cilia (Figure S5D). However, SEM analysis revealed a reduced percentage of ciliated epithelial cells and shortened ciliary length in the tracheobronchial epithelium of hydrocephalic *Kif6* ^M1/M1^ mice compared with controls (Figure S5E–G). Consistently, high‐speed video microscopy showed impaired tracheobronchial ciliary motility in hydrocephalic *Kif6* ^M1/M1^ mice (Figure S5H). These results indicate that hydrocephalic *Kif6* ^M1/M1^ mice exhibit detectable respiratory motile cilia abnormalities, mainly characterized by reduced epithelial ciliation, shortened cilia, and impaired ciliary motility. Whether these ciliary defects predispose mice to respiratory tract infection remains to be determined.

Given the marked reduction in sperm count and increased germ cell apoptosis in *Kif6* mutants, we sought to determine whether KIF6 deficiency might compromise the earlier meiotic phase of spermatogenesis. Analysis of chromosome spreads from prophase I spermatocytes, stained for SYCP3 (a synaptonemal complex component) and γH2AX (marking DNA double‐strand breaks), revealed indistinguishable patterns of homologous chromosome synapsis and recombination between *Kif6* ^M1/M1^ and WT controls (Figure S6A–C). These results indicate that loss of KIF6 does not disrupt normal meiotic progression, and that the asthenozoospermia and infertility observed in *Kif6* mutant mice likely arise from defects during post‐meiotic spermiogenesis, the developmental stage at which KIF6 expression peaks and flagellar assembly occurs. In support of this, PAS staining showed malformed elongating spermatids in stage XI–XII seminiferous tubules of *Kif6* ^M1/M1^ testes (Figure S7A).

### 2.3 KIF6 Is Essential for Sperm Flagellar Motility

**FIGURE 3:**
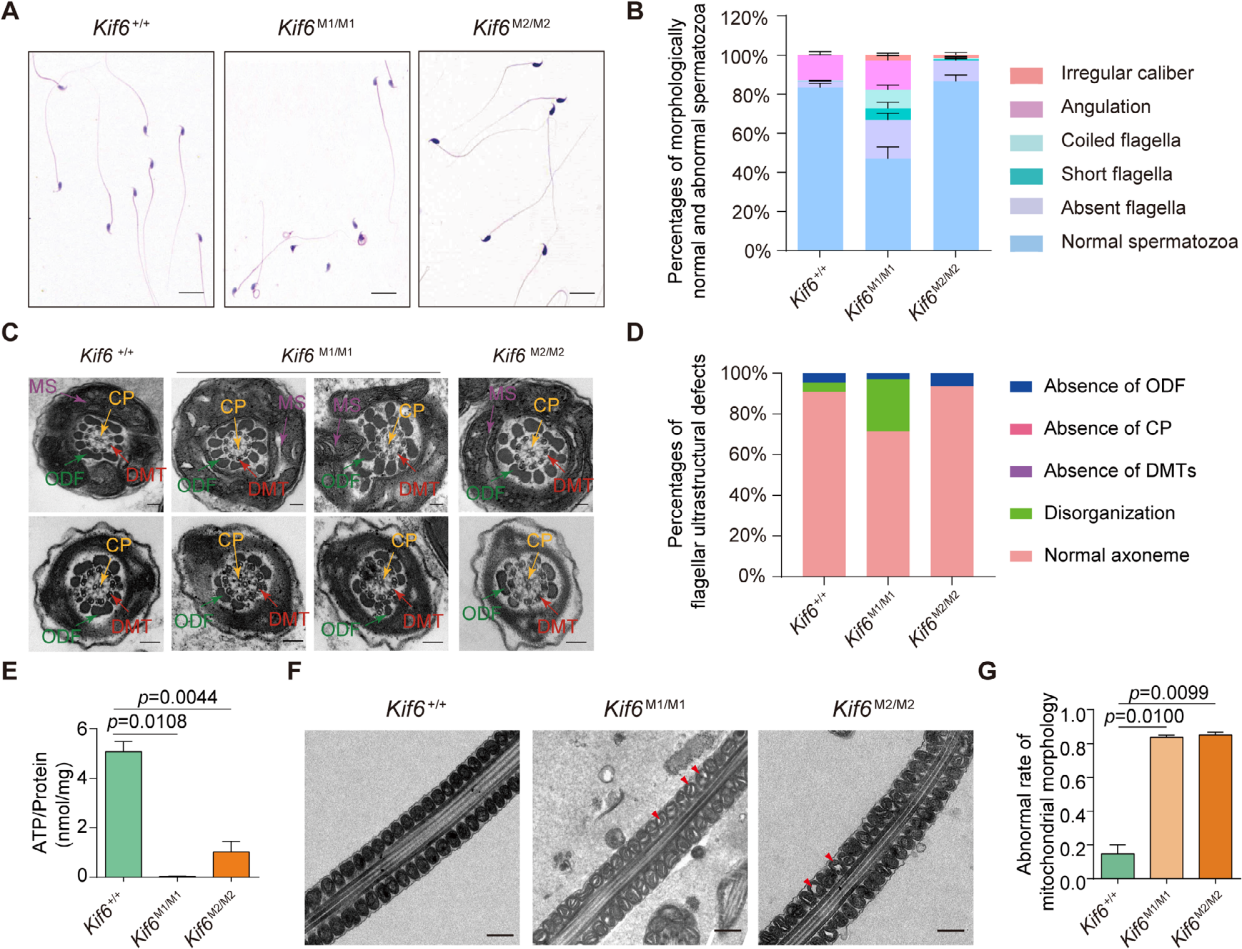
*Kif6* deficiency disrupts flagellar structure and compromises sperm bioenergetics. (A) Representative Papanicolaou staining of caudal sperm from *Kif6* ^+/+^ and *Kif6* mutant mice. Scale bars, 20 µm. (B) Quantification of spermatozoa with normal vs. abnormal flagella in *Kif6* ^+/+^ and *Kif6* mutant mice. Data are mean ± SEM (*n* = 3 mice per genotype). (C) Representative TEM cross‐sections of sperm flagella from *Kif6* ^+/+^ and *Kif6* mutant mice. Key structures are indicated: CP (yellow arrow), MS (purple arrow), DMT (red arrow), and ODF (green arrow). Scale bars, 100 nm. (D) Quantification of distinct categories of flagellar ultrastructural defects in *Kif6* mutant spermatozoa (n = 50 spermatozoa per genotype). (E) ATP levels in caudal sperm from *Kif6* ^+/+^ and *Kif6* mutant mice. Data are mean ± SEM (n = 3 mice per genotype). (F) Representative TEM images of longitudinal sections of sperm flagella from *Kif6* ^+/+^ and *Kif6* mutant mice. Red arrowheads indicate mitochondria with altered internal morphology. Scale bars, 500 nm. (G) Quantification of the proportion of abnormal mitochondria observed in (F) (n = 3 mice per genotype). *P* values were calculated by one‐way ANOVA with Dunnett T3 correction for multiple comparisons (E, G).

The sperm flagellum is the principal organelle responsible for generating propulsive force. To determine whether *Kif6* mutations disrupt flagellar assembly, we examined caudal sperm from mutant and WT mice using Papanicolaou staining and TEM. In *Kif6* ^M1/M1^ males, approximately half of the spermatozoa displayed overt flagellar defects, including shortened or irregular tails, and *Kif6* ^M2/M2^ spermatozoa exhibited largely normal morphology (Figure 3A,B). Consistent with these observations, TEM analysis revealed marked ultrastructural abnormalities in approximately 30% of *Kif6* ^M1/M1^ spermatozoa, characterized by mitochondrial sheath vacuolation and disruption of the canonical “9+2” axonemal structure, and *Kif6* ^M2/M2^ spermatozoa showed no obvious abnormalities in flagellar ultrastructure (Figure 3C,D).

Because a substantial fraction of sperm from both mutants retained normal morphology despite severe motility defects, we next investigated whether underlying functional deficiencies, such as impairments in mitochondrial energy metabolism, might account for the motility defect (Figure 3 and Figure S7B). Given that ATP is the primary energy source for flagellar beating [35], we quantified ATP levels in caudal sperm and observed a significantly reduced ATP content in both *Kif6* ^M1/M1^ and *Kif6* ^M2/M2^ sperm relative to controls (Figure 3E), indicating a compromised mitochondrial bioenergetic output. Consistent with reduced ATP production, TEM analysis revealed defective mitochondrial architecture in both *Kif6* mutant sperm, characterized by vacuolated morphology and disorganized cristae, contrasting with the tightly packed, lamellar cristae in WT sperm (Figure 3F,G), consistent with impaired mitochondrial structural support for energy production. Together, these findings establish KIF6 as an indispensable regulator of sperm flagellar motility.

### 2.4 Multi‐Omics Analyses Reveal that KIF6 Deficiency Remodels Flagellar Components and Metabolic Pathways

**FIGURE 4:**
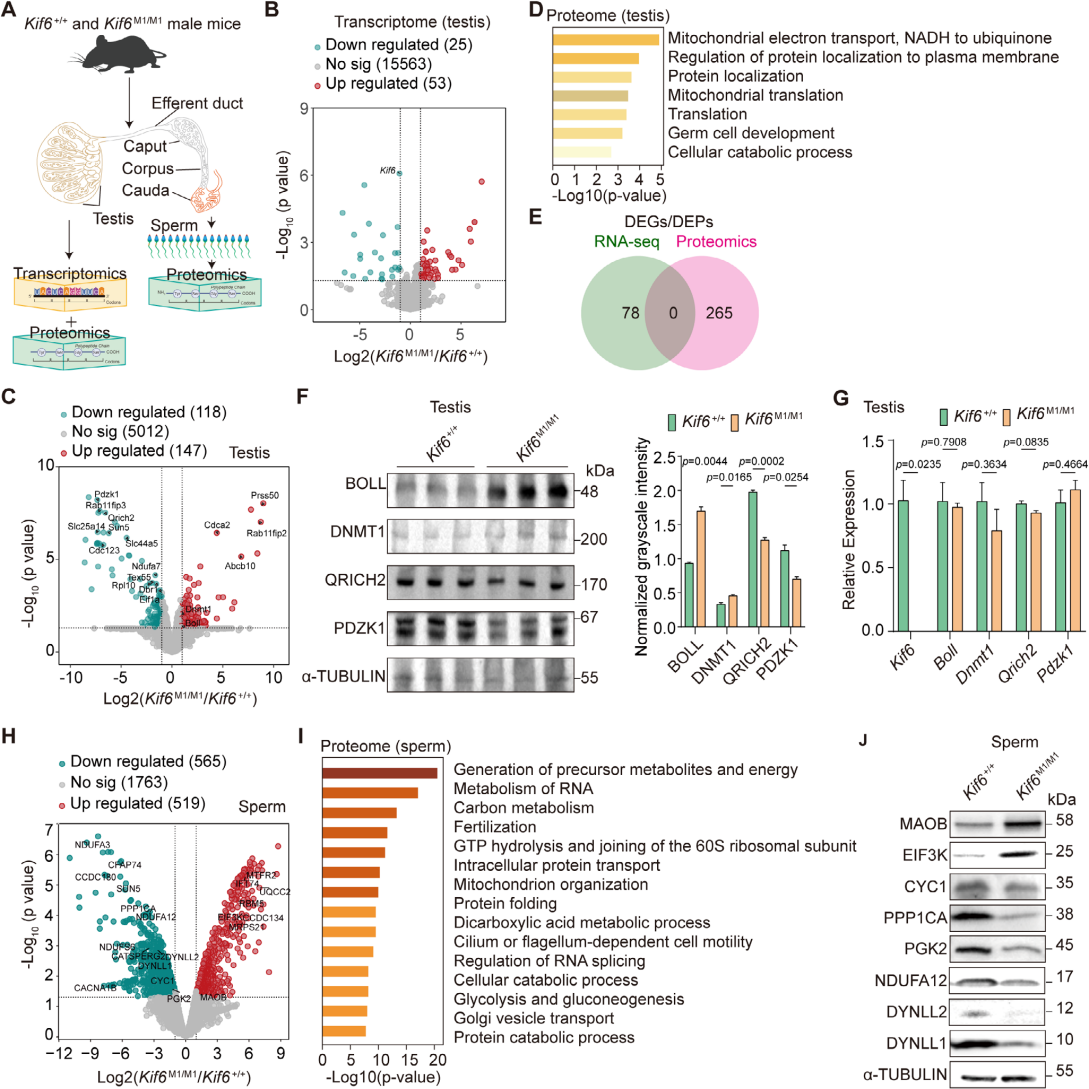
Integrated transcriptomic and proteomic profiling reveals disrupted protein expression programs in *Kif6* ^M1/M1^ testes and sperm. (A) Schematic overview of the experimental design for transcriptomic and proteomic analyses. RNA‐seq and label‐free quantitative mass spectrometry‐based proteomics were performed on adult testes and caudal sperm from *Kif6* ^+/+^ and *Kif6* ^M1/M1^ mice (8–10 weeks old; *n* = 3 mice per genotype). (B) Volcano plot showing RNA‐seq results from adult testes of *Kif6* ^M1/M1^ mice relative to *Kif6* ^+/+^ controls. Differentially expressed genes (DEGs) were identified using DESeq2 with a false discovery rate (FDR) < 0.05. Upregulated genes are shown in red, downregulated genes in green, and non‐significant genes in gray. (C) Volcano plot of proteomic changes in adult testes from *Kif6* ^M1/M1^ mice compared with *Kif6* ^+/+^ controls, based on label‐free quantitative mass spectrometry (n = 3 mice per genotype; No sig, no significant difference). (D) Metascape functional enrichment analysis of differentially expressed proteins (DEPs) in adult testes comparing *Kif6* ^+/+^ and *Kif6* ^M1/M1^ mice. (E) Venn diagram analysis revealed no overlap between DEGs detected at the transcriptomic and proteomic levels in *Kif6* ^M1/M1^ testis. (F) Representative DEPs identified by proteomic profiling of adult testes from *Kif6* ^+/+^ and *Kif6* ^M1/M1^ mice (left), with corresponding Western blot quantification shown on the right. Data are presented as mean ± SEM (n = 3 mice per genotype). (G) RT–qPCR quantification of corresponding mRNA levels for proteins shown in E. Values are normalized to β‐actin (mean ± SEM, *n* = 3 mice per genotype). (H) Volcano plot of proteomics profiling of caudal sperm from *Kif6* ^+/+^ and *Kif6* ^M1/M1^ mice (*n* = 3 mice per genotype; No sig, no significant difference). (I) Metascape enrichment analysis of DEPs identified in caudal epididymis sperm. (J) Representative Western blotting detection of axonemal components (DYNLL1, DYNLL2) and mitochondrial respiratory chain proteins (NDUFA12, CYC1) in caudal sperm. Tubulin served as a loading control. *p*‐values were calculated by unpaired two‐tailed Student's *t*‐test with Welch's correction (F,G).

Given the distinct nature of the two *Kif6* mutant alleles and their divergent sperm phenotypes, we adopted tailored multi‐omics strategies for *Kif6* ^M1/M1^ and *Kif6* ^M2/M2^ mice. The *Kif6* ^M1/M1^ frameshift mutation results in complete loss of KIF6 protein and is associated with a high incidence of flagellar structural abnormalities and reduced sperm count. We therefore focused on testicular transcriptomic and proteomic analyses, together with proteomic profiling of caudal sperm (Figure 4A), to assess how KIF6 loss reshapes flagellar protein composition. Because the high proportion of morphologically abnormal sperm in *Kif6* ^M1/M1^ males is expected to broadly perturb metabolic readouts, metabolomic analysis was not pursued for this model.

RNA sequencing identified only 78 differentially expressed genes (DEGs) (Figure 4B and Data S1). Gene Ontology (GO) analysis of the transcriptomic data revealed no significant enrichment of pathways related to spermatogenesis (Figure S8A), suggesting that KIF6 deficiency exerts limited effects at the transcriptional level. In contrast, proteomic profiling detected 5277 proteins in total, among which 265 were differentially abundant (118 downregulated and 147 upregulated) (Figure 4C and Data S2). Principal component analysis (PCA) demonstrated a clear separation between genotypes (Figure S8B), indicating substantial proteomic alterations. GO analysis highlighted that these altered proteins are predominantly enriched in processes including protein localization, translation, germ cell development, and mitochondrial bioenergetic pathways, with prominent involvement of the mitochondrial electron transport chain (Figure 4D). Notably, integrative analysis of the transcriptomic and proteomic datasets uncovered a discordant regulatory pattern: changes in protein abundance were not accompanied by corresponding alterations in mRNA levels (Figure 4E). Western blotting further showed the altered expression of selected flagellar‐associated proteins encoded by *Boll*, *Dnmt1*, *Qrich2*, and *Pdzk1* (Figure 4F), whereas both RNA‐seq data and RT–qPCR analysis revealed that these transcript levels remained unchanged between *Kif6* ^+/+^ and *Kif6* ^M1/M1^ testes (Figure 4G and Figure S8C). Together, these findings identify transcript–protein discordance as a prominent feature of KIF6 deficiency and suggest that the protein‐level defects in *Kif6* mutant sperm cannot be explained solely by changes in steady‐state mRNA abundance, pointing to a post‐transcriptional regulatory layer.

Proteomic analysis on caudal sperm identified 1,084 differentially abundant proteins among 2,847 proteins detected in *Kif6* ^M1/M1^ sperm (Figure 4H and Figure S8B, and Data S3), including 565 downregulated and 519 upregulated proteins. These proteins are enriched in pathways related to mitochondrial bioenergetic capacity, including precursor metabolite and energy generation, mitochondrial organization, and cilium/flagellum‐dependent cell movement, as well as RNA metabolism and intracellular protein transport (Figure 4I). Western blotting confirmed changes in representative structural and metabolic proteins (e.g., DYNLL1/2, NDUFA12, CYC1) (Figure 4J). Combined with the observed axonemal and mitochondrial ultrastructural defects and the marked reduction in ATP levels, these findings indicate that KIF6 is essential for maintaining flagellar proteome homeostasis and mitochondrial bioenergetics.

**FIGURE 5:**
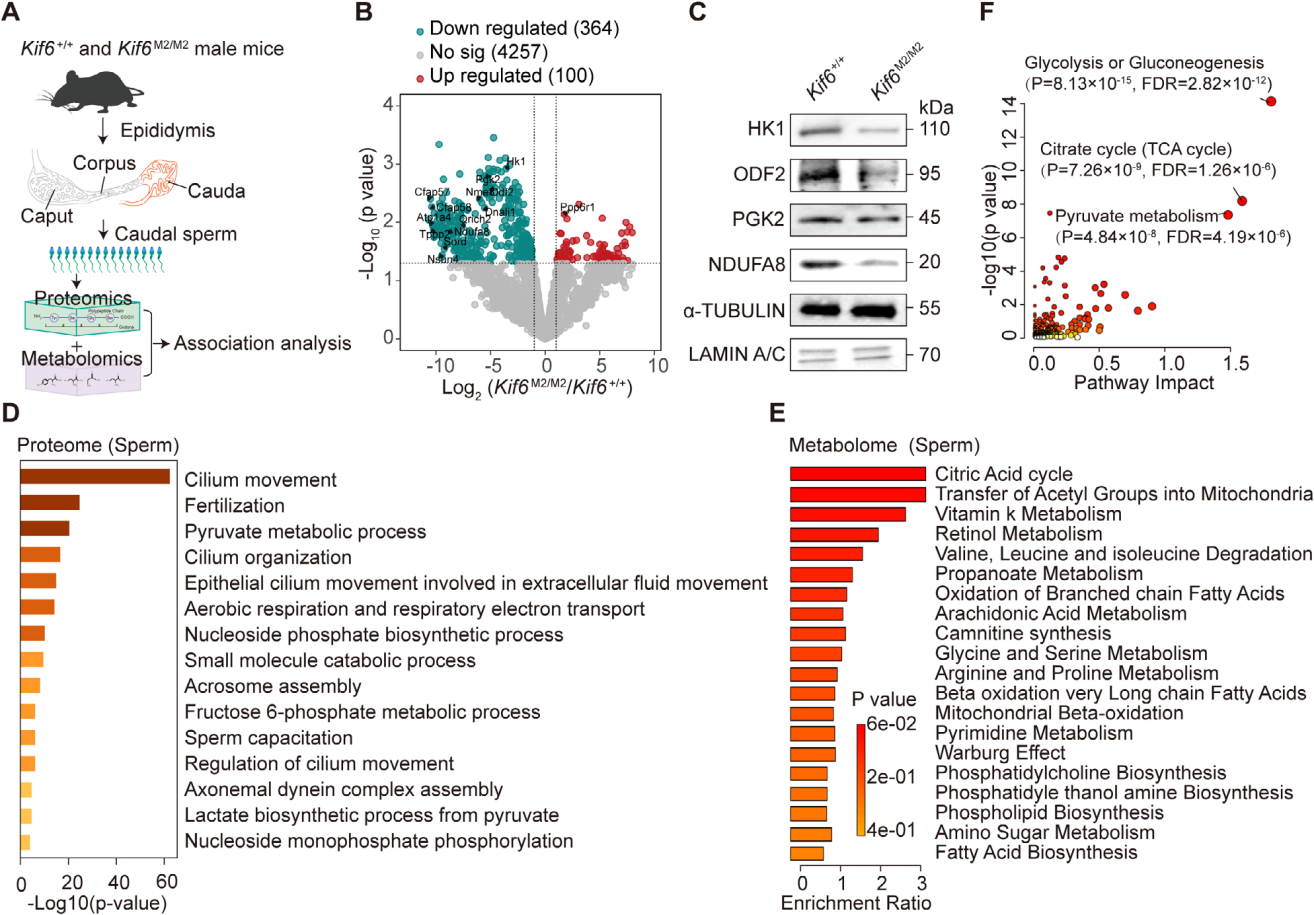
Integrated proteomic and metabolomic analyses reveal impaired energy metabolism in *Kif6* ^M2/M2^ sperm. (A) Schematic overview of the experimental design for integrated proteomic and metabolomic profiling. Proteomic and metabolomic analyses were performed on caudal sperm from adult *Kif6* ^+/+^ and *Kif6* ^M2/M2^ mice. (B) Volcano plot of DEPs identified in caudal sperm from *Kif6* ^M2/M2^ relative to *Kif6* ^+/+^ controls, based on mass‐spectrometry‐based proteomics (n  =  3 mice per genotype). Upregulated proteins are shown in red, downregulated proteins in green, and non‐significant proteins in gray. (C) Representative examples of DEPs in *Kif6* ^M2/M2^ sperm relative to controls. (D) Metascape functional enrichment analysis of DEPs identified in sperm from *Kif6* ^+/+^ and *Kif6* ^M2/M2^ mice. (E) Pathway‐level differences in metabolite abundance between sperm from *Kif6* ^+/+^ and *Kif6* ^M2/M2^ mice. Metabolite pathways sourced from the KEGG database. (F) Integrated proteome‐metabolome association analysis performed using MetaboAnalyst 6.0, showing relationships between differentially abundant proteins and metabolites in caudal sperm from *Kif6* ^+/+^ and *Kif6* ^M2/M2^ mice.

In contrast, *Kif6* ^M2/M2^ spermatozoa retain largely intact flagellar ultrastructure but exhibit pronounced motility defects, suggesting a functional rather than structural origin. Accordingly, we performed integrated proteomic and metabolomic analyses on caudal sperm from *Kif6* ^M2/M2^ mice to investigate whether altered energy metabolism and mitochondrial function underlie the motility impairment (Figure 5A). Proteomic analysis revealed 464 differentially expressed proteins (364 downregulated and 100 upregulated) (Figure 5B,C and Figure S8D and Data S4). GO analysis showed enrichment in processes including cilium movement, pyruvate metabolism, cilium organization, aerobic respiration, and mitochondrial electron transport (Figure 5D). Metabolomic KEGG analysis further revealed perturbations in energy and redox metabolism pathways, including citric acid cycle, transfer of acetyl groups into mitochondria, Warburg effect, and fatty acid metabolism (Figure 5E and Data S5). Integrated pathway analysis revealed disruptions in both glycolysis and mitochondrial oxidative phosphorylation in *Kif6*‐deficient sperm (Figure 5F), further supporting the compromised mitochondrial metabolic capacity in these sperm.

Taken together, these multi‐omics analyses demonstrate that loss of KIF6 results in profound remodeling of the sperm flagellar proteome and metabolic pathways, despite only minimal alterations at the transcriptomic level. Although the two *Kif6* mutant alleles give rise to distinctly different sperm phenotypes, they are both characterized by impaired protein composition and bioenergetic capacity within the flagellum. These findings suggest that KIF6 plays an essential role in maintaining the delivery or stabilization of axonemal and metabolic components, which are critical prerequisites for flagellar motility.

### 2.5 KIF6 Interacts With RNA‐Binding Proteins FMRP and FXR1

**FIGURE 6:**
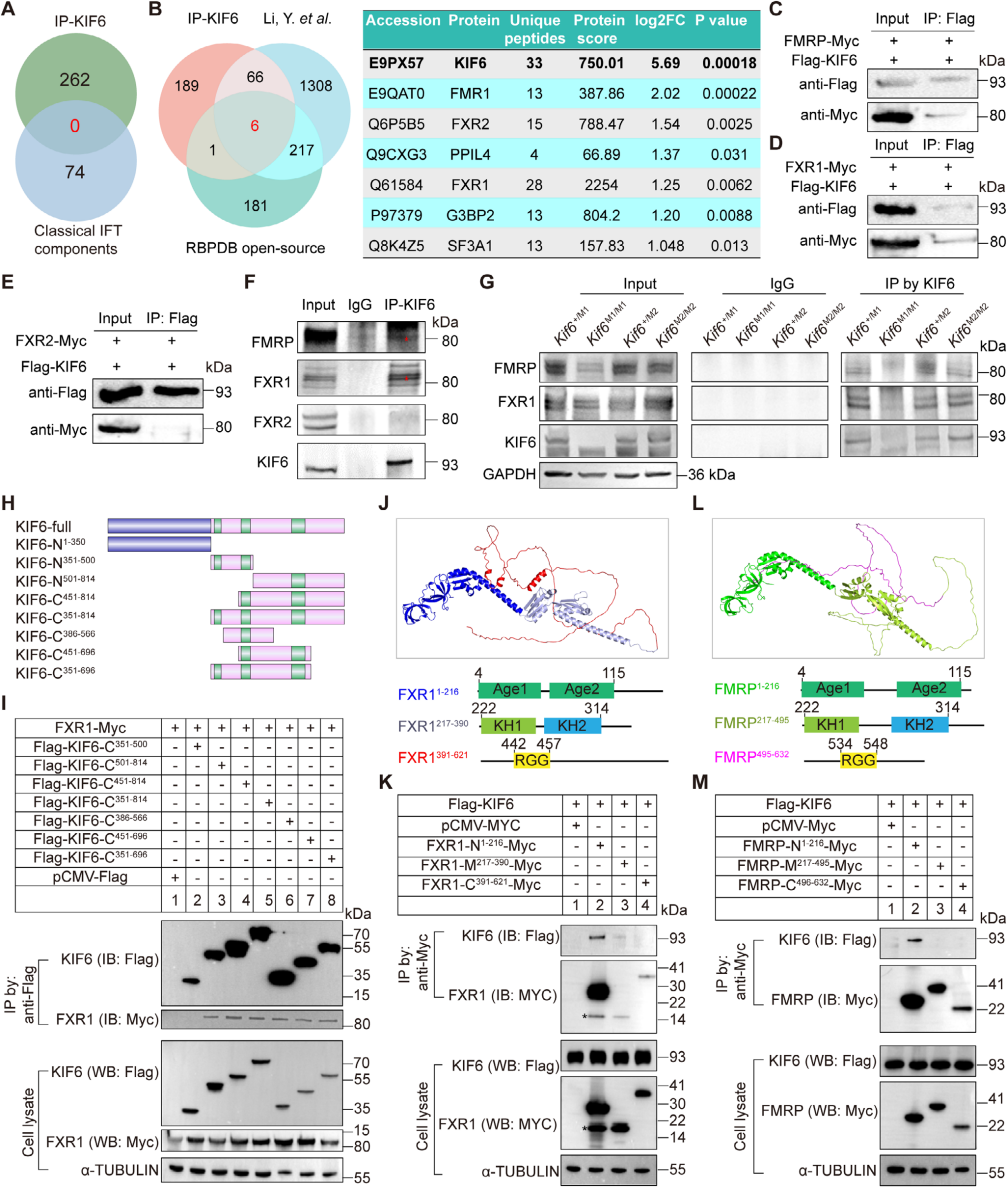
KIF6 interacts with the RBPs FMRP and FXR1. (A) Overlap analysis between KIF6‐associated proteins identified by IP–MS (262 significantly enriched proteins) and annotated canonical IFT components. (B) Intersection analysis between the 262 KIF6‐associated proteins identified by IP–MS and curated RBP databases. (C–E) Co‐IP analysis of epitope‐tagged KIF6 with FMRP (C), FXR1 (D), or FXR2 (E) in HEK293T cells. (F) Endogenous co‐IP analysis using WT mouse testicular lysates to examine KIF6 association with FMRP and FXR1. Red asterisks indicate specific immunoreactive bands. (G) Co‐IP analysis of testicular lysates from *Kif6* ^M1/M1^ and *Kif6* ^M2/M2^ mice. Immunoprecipitation was performed using anti‐KIF6 antibodies, followed by immunoblotting for KIF6, FMRP and FXR1. (H) Schematic representation of full‐length KIF6 and a series of deletion constructs used for domain‐mapping analyses. (I) Co‐IP analysis of KIF6 truncation constructs expressed in HEK293T cells, assessing association with FXR1. (J) Predicted structural model of FXR1 retrieved from the AlphaFold Protein Structure Database (AF‐Q2TBT7‐F1‐v6; top), and schematic representation of FXR1 truncation constructs corresponding to N‐terminal, middle, and C‐terminal regions (bottom). (K) Co‐IP analysis of FXR1 truncation constructs expressed in HEK293T cells, assessing association with full‐length KIF6. Black asterisks denote non‐specific bands. (L) Predicted structural model of FMRP retrieved from the AlphaFold Protein Structure Database (AF‐Q06787‐F1‐v6; top), and schematic representation of FMRP truncation constructs corresponding to N‐terminal, middle, and C‐terminal regions (bottom). (M) Co‐IP analysis of FMRP truncation constructs expressed in HEK293T cells, assessing association with full‐length KIF6.

To validate the above hypothesis and elucidate the molecular mechanism underlying KIF6‐dependent flagellar motility and function, we performed co‐immunoprecipitation followed by mass spectrometry (IP–MS) using a KIF6‐specific antibody on WT mouse testicular lysates, with non‐specific IgG as a negative control. In total, 262 proteins were significantly enriched in KIF6 immunoprecipitates (*p*< 0.05, log2 fold change > 1) (Data S6). Cross‐referencing these candidates with annotated IFT components, as defined previously [36, 37], revealed no overlap, indicating that KIF6 does not function through the canonical IFT machinery (Figure 6A). GO analysis of these interacting proteins showed significant enrichment in pathways related to microtubule binding, mRNA binding, and translational regulation (Figure S9A).

We further noted that RBPs constituted a substantial proportion of the 262 enriched proteins, consistent with the GO enrichment for mRNA‐binding functions and the well‐established role of RBPs in regulating the translation of stored mRNAs during spermiogenesis [38]. To further examine the RBP component of the KIF6 interactome, we cross‐referenced the 262 candidate interactors with curated RBP databases [38, 39], which identified six spermatogenesis‐ associated RBPs (Figure 6B). Among these, FMRP, FXR1, and FXR2–key components of a conserved ribonucleoprotein complex involved in mRNA transport and translational regulation–were prioritized for validation [40, 41, 42]. Co‐immunoprecipitation (co‐IP) assays demonstrated that KIF6 interacts with both FMRP and FXR1 under exogenous expression conditions (Figure 6C–E), and endogenous interactions were verified by IP in WT testicular lysates (Figure 6F).

To further assess whether KIF6 preferentially associates with FMRP or FXR1, we performed reciprocal competition co‐IP assays. FXR1 recovery in KIF6 immunoprecipitates was not substantially reduced by increasing FMRP, whereas FMRP recovery was progressively reduced by increasing FXR1, suggesting that FXR1 displays a stronger competitive association with KIF6 under these assay conditions (Figure S10A,B). Immunofluorescence analysis further showed that FMRP and FXR1 exhibited overlapping but distinct expression patterns during spermatogenesis: FMRP was highly expressed in spermatocytes and remained detectable during spermiogenesis, whereas FXR1 was also detected in spermatocytes and further increased during spermiogenesis (Figure S10C,D).

We next assessed the effects of pathogenic *Kif6* mutations on these interactions. In *Kif6* ^M1/M1^ mice, co‐IP assays detected no KIF6–FMRP or KIF6–FXR1 complexes (Figure 6G), consistent with the loss of detectable KIF6 expression and disruption of KIF6‐dependent complex assembly. In contrast, *Kif6* ^M2/M2^ testicular lysates retained detectable KIF6–FMRP/FXR1 complexes (Figure 6G).

To define the structural basis of the KIF6–RBP interaction, we generated N‐terminal and C‐terminal truncation mutants of KIF6. Co‐IP assays revealed that both the N‐terminal motor domain and the C‐terminal coiled‐coil domain of KIF6 interact with FMRP and FXR1 (Figure S9B,C). Since the C‐terminal domain of kinesins typically mediates cargo binding [43], we further generated a series of C‐terminal truncation mutants and mapped the minimal FXR1‐binding region to residues 386–566 (Figure 6H,I), a segment that encompasses the M2 missense mutation site. Reciprocally, we found that the N‐terminal domains of both FXR1 and FMRP, which share high sequence homology (Figure S9D), are necessary for interaction with KIF6 (Figure 6J–M).

Further biochemical analyses demonstrated that KIF6 can self‐associate, as shown by co‐IP of differently tagged full‐length KIF6 proteins co‐expressed in HEK293T cells (Figure S9E). Since processive microtubule‐based movement typically requires kinesins to function as oligomers rather than monomers [14], this self‐association suggests that KIF6 possesses the intrinsic capacity to mediate microtubule‐dependent motility. Additionally, in vitro assays confirmed an intramolecular interaction between the N‐terminal and C‐terminal domains of KIF6 (Figure S9F,G), supporting the previously reported autoinhibitory function of KIF6 [34]. Notably, KIF9, the other kinesin‐9 family member, did not detectably interact with FMRP or FXR1 under the same co‐IP conditions (Figure S9H,I), supporting the functional specificity of KIF6 in RBP‐mediated mRNA transport.

Collectively, these results indicate that KIF6 participates in the regulation of sperm flagellar motility and function by forming a complex with the RBPs FMRP and FXR1, and they identify amino acid residues 386–566 as a key region mediating KIF6–RBP interactions.

### 2.6 KIF6‐RBP Complex Mediates mRNA Transport Required for Flagellar Biogenesis

**FIGURE 7:**
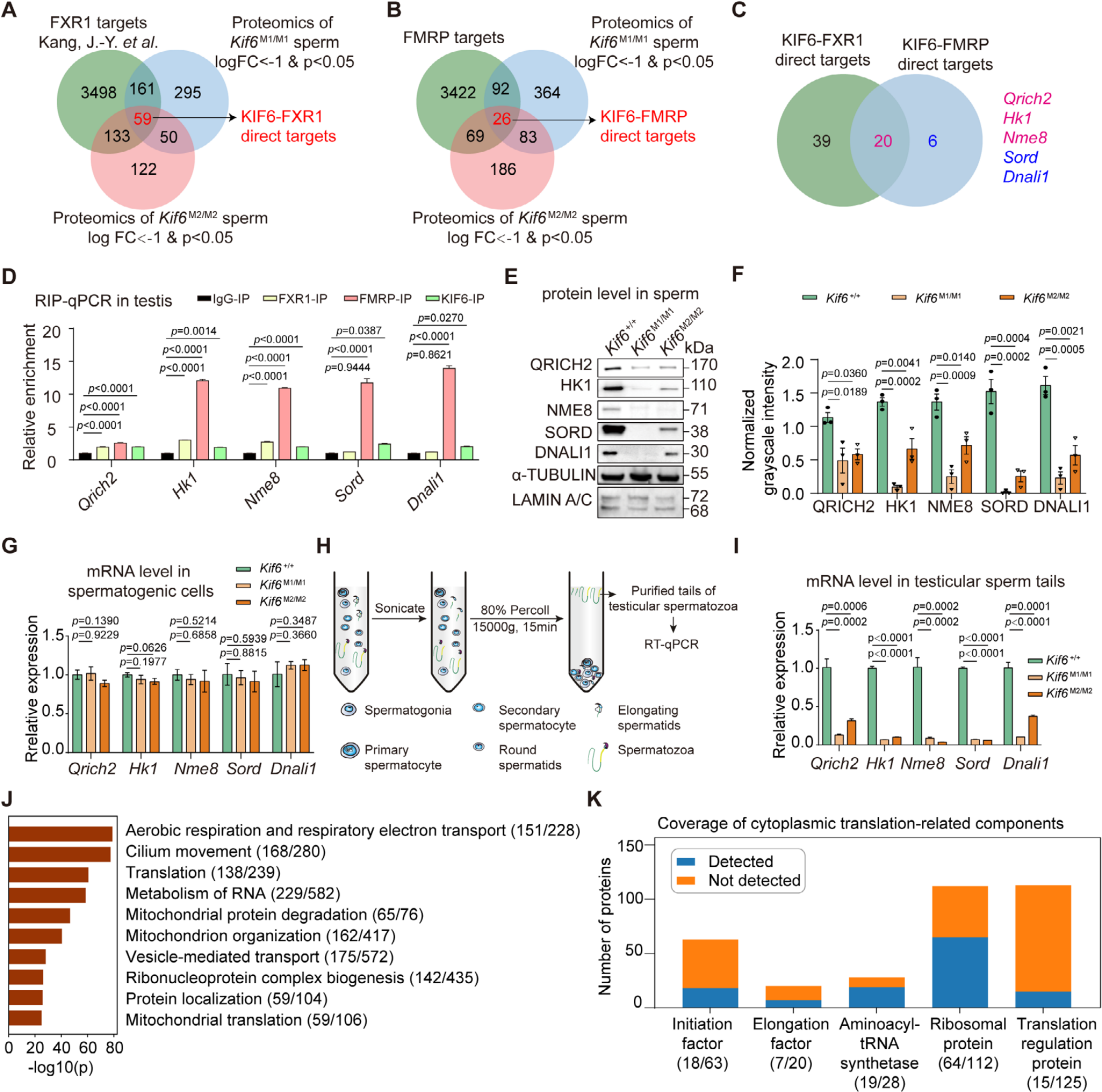
KIF6 cooperates with FMRP and FXR1 to mediate selective transport of mRNAs required for flagellar biogenesis. (A) Intersection analysis between previously reported FXR1‐bound transcripts (as defined by log2 fold change > 1 and FDR < 0.05) and proteins showing reduced abundance in sperm from *Kif6* ^M1/M1^ and *Kif6* ^M2/M2^ mice. A total of 59 transcripts were identified for subsequent analysis. (B) Intersection analysis of 3,609 high‐confidence FMRP‐associated transcripts with proteins showing reduced abundance in sperm from *Kif6* ^M1/M1^ and *Kif6* ^M2/M2^ mice. A total of 26 transcripts were identified for subsequent analysis. (C) Venn diagram depicting the overlap between transcript sets identified in (A) and (B). Shared transcripts (*Qrich2*, *Hk1*, *Nme*8) and transcripts identified only in the FMRP‐associated set (*Sord*, *Dnali1*) were selected for downstream analyses. (D) RIP‐qPCR performed on adult mice testes to assess the association of selected transcripts with KIF6, FMRP, and FXR1. Data are presented as mean ± SEM (*n* = 3 mice per genotype). (E) Representative Western blot analysis of proteins encoded by selected transcripts in caudal sperm from *Kif6* ^+/+^, *Kif6* ^M1/M1^, and *Kif6* ^M2/M2^ mice. LAMIN A/C served as a loading control. (F) Quantification of Western blot analysis shown in E. Data are presented as mean ± SEM (n  =  3 mice per genotype). (G) RT–qPCR analysis of mRNA abundance for selected transcripts in adult testes from *Kif6* ^M1/M1^ and *Kif6* ^M2/M2^ mice. Data are presented as mean ± SEM (*n* = 3 mice per genotype). (H) Schematic overview of the workflow used for isolation of flagella from testicular spermatids. (I) RT–qPCR analysis of selected transcripts in purified flagellar fractions from testicular spermatids of WT, *Kif6* ^M1/M1^, and *Kif6* ^M2/M2^ mice. Data are presented as mean ± SEM (*n* = 3 mice per genotype). (J) Pathway enrichment analysis of proteins identified by proteomic profiling of enriched mature sperm flagella, performed using Metascape. Numbers in parentheses denote the number of detected proteins vs. the total number of proteins annotated to each pathway. (K) Detection profile of cytoplasmic translation‐related components within the flagellar proteome. *P* values were calculated by one‐way ANOVA with Dunnett's multiple comparisons test (D, F, G, I).

To identify the direct mRNA cargoes of the KIF6–FXR1 complex, we performed an overlap analysis between previously reported FXR1‐bound transcripts [44] and downregulated proteins in the sperm proteomes of *Kif6* ^M1/M1^ and *Kif6* ^M2/M2^ males. A total of 59 potential direct targets of the KIF6–FXR1 complex were identified (Figure 7A and Data S7). Functional enrichment analysis revealed that these targets are significantly associated with cilium movement (e.g., *Ccdc38* linked to flagellar biogenesis [45], as well as *Armc12* and *Qrich2* related to mitochondrial function [46, 47]), succinate metabolism, and carbon metabolism pathways (Figure S11A).

To characterize the mRNA cargoes of the KIF6–FMRP complex, we performed RNA immunoprecipitation sequencing (RIP‐seq) using an FMRP‐specific antibody in adult mouse testes. This analysis identified 4788 FMRP‐bound transcripts (Figure S11B), and 3609 high‐confidence FMRP targets were obtained following stringent quality control (Data S8). FMRP‐binding sites were predominantly localized within the coding regions of target mRNAs (Figure S11C), consistent with its established role in translational regulation. Functional enrichment analysis showed that these FMRP targets are significantly enriched in pathways closely related to spermatogenesis and flagellar motility, including male gamete generation, microtubule cytoskeleton organization, microtubule‐based transport, and regulation of mRNA metabolism (Figure S11D). To further define direct cargoes of the KIF6–FMRP complex, we intersected these 3609 FMRP target mRNAs with proteins downregulated in *Kif6*‐mutant sperm, yielding 26 core candidate mRNAs (Figure 7B and Data S7). These candidates were significantly enriched in cilium movement and carbohydrate metabolism pathways (Figure S11E).

Further analysis revealed 20 shared targets between the KIF6–FMRP and KIF6–FXR1 complexes. Given the well‐documented mRNA‐binding profile of FXR1 in previous studies, we selected five direct molecular targets of the KIF6–FMRP complex for in‐depth validation, including two unique targets (*Sord*, involved in energy metabolism [48], and *Dnali1*, encoding an inner dynein arm component [49]) and three shared targets (*Qrich2*, implicated in flagellar biogenesis and mitochondrial function [46]; *Hk1*, encoding a key metabolic enzyme [50, 51]; and *Nme8*, associated with sperm motility [52]) (Figure 7C). RNA immunoprecipitation combined with quantitative PCR (RIP‐qPCR) confirmed that KIF6–FMRP binds all five candidate transcripts, while KIF6–FXR1 also interacts with the three shared targets (Figure 7D). These results demonstrate that KIF6 forms functional complexes with FMRP and FXR1 to selectively transport specific mRNA cargoes.

Subsequently, we validated protein expression by Western blotting and mRNA abundance by RT–qPCR. Consistent with previous proteomic analysis, all five proteins were significantly downregulated in sperm from both *Kif6* ^M1/M1^ and *Kif6* ^M2/M2^ mice compared with WT controls (Figure 7E,F). Importantly, the transcript levels of these targets in the testes of mutant mice remained unchanged (Figure 7G). We purified flagella from testicular spermatids of WT and *Kif6*‐mutant mice (Figure 7H and Figure S11F). RT‐qPCR analysis of the purified flagella showed that the enrichment of validated target mRNAs in the flagellum was significantly reduced in both mutants compared to WT (Figure 7I). Together with the aforementioned results, the reduced mRNA levels in the flagellum are likely attributed to impaired mRNA transport capacity caused by *Kif6* mutations, further supporting that KIF6–RBP complexes mediate the transport of mRNAs required for flagellar biogenesis.

To investigate whether key components of local translation are present in sperm flagella, we first constructed a reference dataset of mouse cytoplasmic translation‐related proteins using annotation information from the Universal Protein Resource (UniProt) associated with relevant GO terms. This dataset was classified into two major categories: 223 core translation machinery components (including ribosomal proteins, translation initiation/elongation factors, and aminoacyl‐tRNA synthetases) and 125 translation regulatory proteins (Data S9). We then enriched mature sperm flagellum samples, and proteomic analysis showed significant enrichment of pathways related to translation, RNA metabolism, and ribonucleoprotein complex biogenesis (Figure 7J). Further analysis demonstrated that we systematically identified core translation machinery components covering all major functional modules of the translational apparatus in the flagellar proteome, including translation initiation factors, elongation factors, ribosomal subunits, and aminoacyl‐tRNA synthetases (Figure 7K and Figure S12, and Data S9). These results suggest the presence of a local translation system in the flagellum. To further assess nascent peptide synthesis in sperm flagella, we performed puromycin incorporation assays in live epididymal sperm from WT, *Kif6* ^M1/M1^, and *Kif6* ^M2/M2^ mice. In WT sperm, puromycin treatment induced a clear puromycylated peptide signal along the flagellum, whereas this signal was strongly reduced by cycloheximide treatment, confirming the specificity of the assay (Figure S13A,B). In contrast, sperm from both *Kif6* ^M1/M1^ and *Kif6* ^M2/M2^mice showed markedly reduced flagellar puromycin signals compared with WT sperm (Figure S13A,B). These results indicate that nascent peptide synthesis activity is detectable in mature sperm flagella and is impaired in *Kif6* mutant sperm.

Taken together, these results support a model in which KIF6–RBP complexes mediate the targeted transport of mRNAs to the flagellum, where these transcripts may contribute to local protein production and flagellar function.

### 2.7 KIF6 Is Essential for mRNA Transport During Sperm Flagellar Biogenesis

Previous studies have established that KIF6–RBP complexes selectively transport mRNAs required for flagellar biogenesis. To further evaluate the functional necessity of KIF6 in mediating the directed mRNP delivery pathway during sperm flagellar biogenesis, and to elucidate how *Kif6* mutations disrupt this process, we selected *Hk1* (a key gene involved in energy metabolism) and *Dnali1* (encoding an inner dynein arm protein) as representative mRNA cargoes and systematically analyzed their subcellular trafficking in elongating spermatids.

**FIGURE 8:**
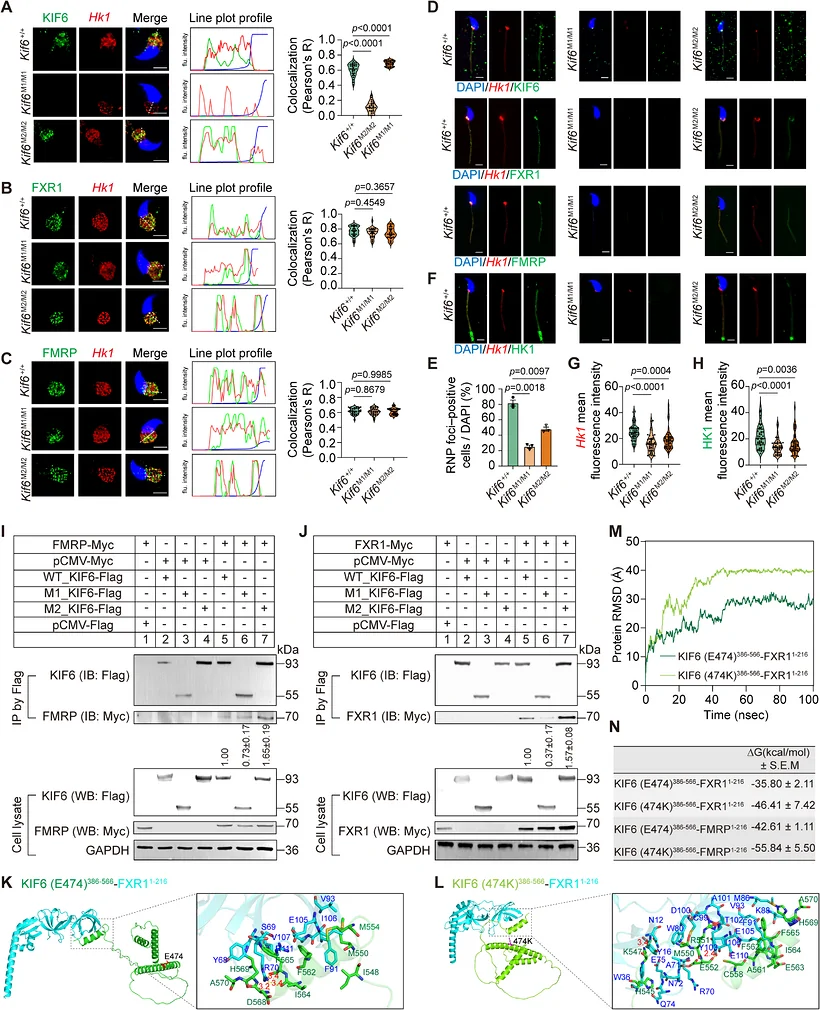
Divergent *Kif6* mutations impair mRNP transport in elongating spermatids by altering the KIF6–FMRP/FXR1 complex. (A–C) Immuno‐FISH of KIF6 (A), FXR1 (B), or FMRP (C) proteins (green) with *Hk1* mRNA (red) in isolated elongating spermatids from *Kif6* ^+/+^, *Kif6* ^M1/M1^, and *Kif6* ^M2/M2^ mice. Representative images (left), line‐scan intensity profiles (middle), and co‐localization quantification (right; n = 30 spermatids per genotype). (D) Representative immuno‐FISH showing subcellular distribution of *Hk1* RNP signals. (E) Percentage of spermatids with *Hk1* RNP signals at the connecting piece (mean ± SEM; n = 3 mice per genotype; 100 spermatids per mouse). (F) Immuno‐FISH of *Hk1* mRNA (red) and HK1 protein (green) in spermatids of the indicated genotypes. (G,H) Quantification of *Hk1* mRNA (G) and HK1 protein (H) fluorescence intensity along the flagellar compartment in elongating spermatids (mean ± SEM; n = 50 spermatids per genotype). (I,J) Co‐IP of Flag–KIF6 (WT or mutants) with Myc–FMRP (I) or Myc–FXR1 (J) in HEK293T cells; GAPDH, loading control; representative blots from three independent experiments with relative band intensities. (K–N) Molecular docking models of the KIF6–FXR1 interface for WT (K) and mutant (L) complexes, RMSD analysis (M), and predicted binding free energies for KIF6(386–566) in complex with FMRP(1–216) or FXR1(1–216) (N; ZDOCK with HawkDock estimation). Scale bars, 5 µm (A–F). In A–C, each dot represents the percentage of protein signal co‐localizing with mRNA signal in an individual spermatid; in G,H, each dot represents the mean flagellar fluorescence intensity from a single spermatid. A–C, G–H, n = 3 biologically independent experiments. *P* values were calculated by one‐way ANOVA with Dunnett T3 correction for multiple comparisons (A–C, E, G, H).

Immunofluorescence in situ hybridization (Immuno‐FISH) revealed that, in the cytoplasm of WT spermatids, *Hk1* mRNA colocalized with KIF6, FMRP, and FXR1 (Figure 8A–C), whereas *Dnali1* mRNA colocalized with KIF6 and FMRP but not with FXR1 (Figure S14A–C). This distribution pattern was highly consistent with the target‐binding specificity validated by RIP‐qPCR. Because *Kif6* ^M1/M1^ carries a frameshift mutation, KIF6 protein was undetectable in spermatids, precluding analysis of KIF6–mRNA colocalization in this mutant. In contrast, in *Kif6* ^M2/M2^ missense mutant spermatids, colocalization of KIF6 with both transcripts in the cytoplasmic region of the sperm head was significantly increased (Figure 8A and Figure S14A), suggesting enhanced retention of KIF6 on mRNP granules. Notably, neither mutation altered the colocalization patterns of FMRP or FXR1 with their cognate mRNAs (Figure 8B,C and Figure S14B,C). These observations indicate that KIF6 acts downstream of mRNP assembly and functions to transport pre‐assembled RBP–mRNA complexes rather than participating in their formation.

The connecting piece is thought to serve as a critical entry site for cargos destined for the developing flagellum. To determine where mRNP transport fails in the absence of functional KIF6, we next examined transcript targeting to the connecting piece. Compared with WT spermatids, both *Kif6* mutant models exhibited a marked reduction in the proportion of spermatids displaying *Dnali1* or *Hk1* mRNA foci at the connecting piece (Figure 8D,E and Figure S14D,E), indicating that KIF6 dysfunction impairs cytoplasmic‐to–connecting‐piece accumulation of these transcripts. Although KIF6, FMRP, and FXR1 colocalized with *Dnali1* or *Hk1* mRNAs at the connecting piece in WT spermatids (Figure 8D and Figure S14D), the corresponding proteins DNALI1 and HK1 were not detected at this site but instead localized specifically along the entire flagellum (Figure 8F and Figure S14F). Importantly, both *Kif6* mutations resulted in a pronounced reduction of these transcripts within the flagellum, accompanied by a corresponding decrease in DNALI1 and HK1 protein abundance along the flagellum (Figure 8G,H and Figure S14G,H). These results suggest that the flagellar localization of KIF6‐RBP target mRNAs is an essential prerequisite for ensuring sufficient protein supply during flagellar assembly.

To elucidate the molecular basis of the enhanced mRNP association observed in the missense mutant, we performed biochemical and structural analyses of KIF6–RBP interactions. Co‐IP assays showed that the E474K substitution increased KIF6 binding to FMRP by 1.65‐fold and to FXR1 by 1.57‐fold (Figure 8I,J). Structural modeling revealed that this mutation remodels the KIF6–RBP interaction interface, introducing new hydrogen bond networks and expanding close‐range contact surfaces between KIF6 and its binding partners (Figure 8K–M and Figure S14I–K). Consistent with these predicted structural changes, binding free‐energy calculations supported a potential increase in the interaction propensity of the mutant protein with both RBPs (Figure 8N).

Collectively, these results demonstrate that KIF6 is a central regulatory factor required for directing specific mRNP cargoes to the connecting piece and for their subsequent enrichment within the flagellum. Distinct *Kif6* mutations disrupt this transport pathway through different mechanisms: the frameshift mutation abolishes KIF6 protein expression and thereby directly blocks mRNP targeting, whereas the missense mutation enhances KIF6–mRNP binding, leading to cytoplasmic retention of transport complexes, impaired recruitment to the connecting piece, and ultimately reduced mRNA delivery into the flagellum (Figure S14L). These findings establish that KIF6, through functional interactions with FMRP or FXR1, mediates the directed transport of specific mRNAs during sperm flagellar biogenesis—a process that is essential for proper mRNA enrichment within the connecting piece and flagellum and for normal sperm flagellar assembly.

## 3 Discussion

**FIGURE 9:**
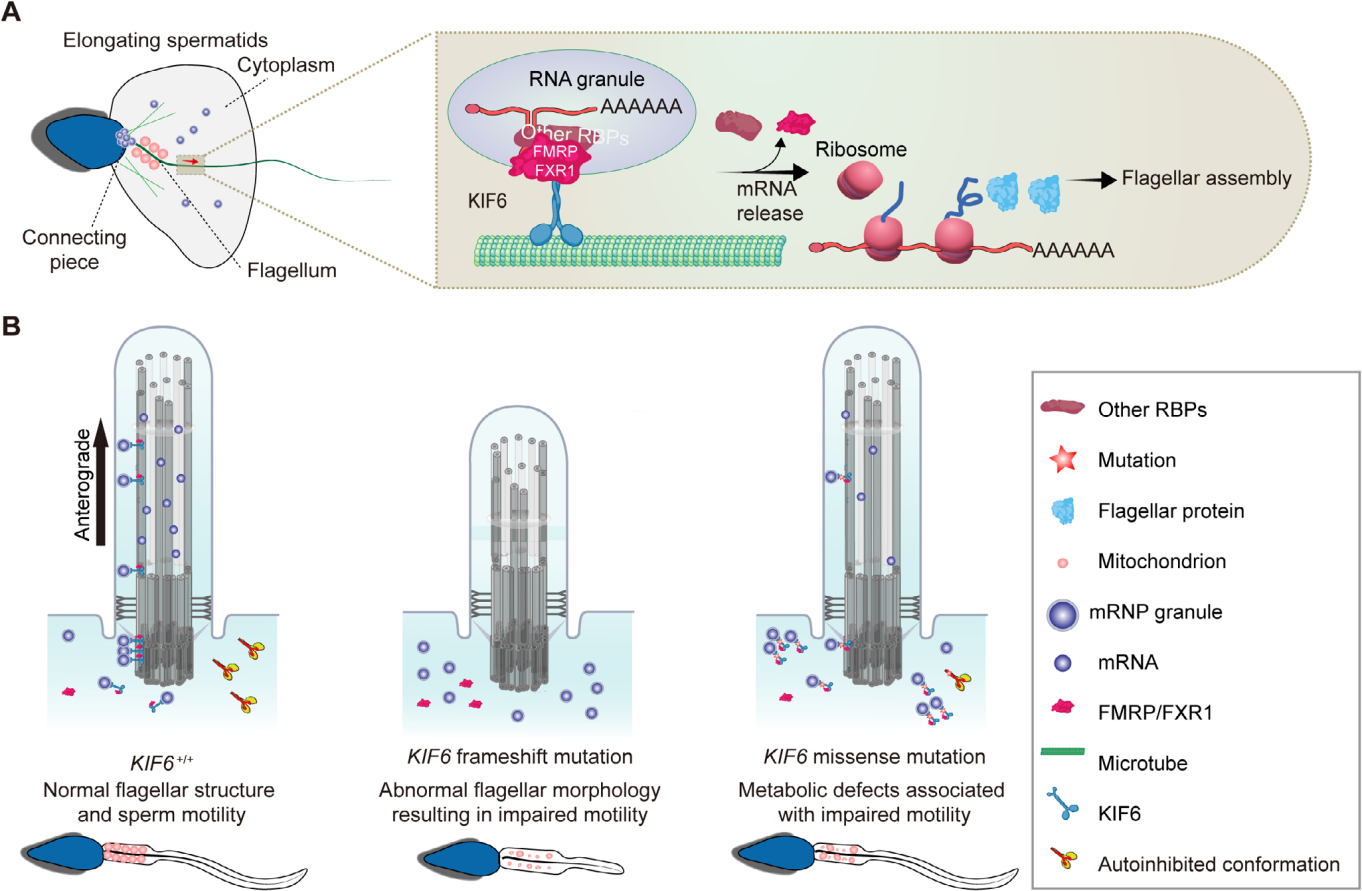
Proposed model for KIF6‐RBP‐mediated mRNA transport during flagellar assembly and the functional defects of *KIF6* deleterious mutations. (A) Proposed model illustrating KIF6‐RBP‐mediated mRNA transport during flagellar assembly. In elongating spermatids, KIF6 associates with FMRP/FXR1‐containing mRNP granules in the cytoplasm and promotes their accumulation at the connecting piece and their subsequent entry into the developing flagellum. (B) Functional defects caused by *KIF6* deleterious mutations. Bottom left (WT): KIF6 facilitates the proper localization of RBP–mRNA granules to the connecting piece and their subsequent presence within developing flagella. This ensures adequate flagellar enrichment of transcripts encoding axonemal components and metabolic enzymes, thereby supporting normal flagellar assembly and sperm motility. Bottom middle (Frameshift mutation, T442Sfs*3): The frameshift mutation results in complete loss of KIF6 expression. Without KIF6, RBP–mRNA granules show markedly reduced accumulation at the connecting piece, leading to diminished entry of KIF6‐dependent transcripts into the developing flagella. This deficit in key structural and metabolic components is consistent with severe axonemal disorganization, disrupted mitochondrial morphology, and complete sperm immotility. Bottom right (Missense mutation, p.E474K): In spermatids carrying the missense mutation, enhanced binding affinity between KIF6 and RBPs is proposed to alter the subcellular distribution of KIF6–mRNP complexes, resulting in increased cytoplasmic retention and reduced localization at the connecting piece and within the flagellum.

Precise assembly of the sperm flagellum is a fundamental prerequisite for successful reproduction in sexually reproducing organisms, including humans, and its molecular regulation has long remained a central question in reproductive biology. For decades, flagellar biogenesis was thought to rely predominantly on the canonical IFT pathway [12]. However, the detection of mRNAs within cilia and flagella [25, 30, 32] has raised the possibility of a non‐canonical flagellar transport mode, distinct from classical IFT, whereby mRNAs themselves act as transport cargo. By integrating human genetic screening, gene‐edited mouse models, and multi‐omics analyses, our study identifies the kinesin‐9 family member KIF6 as an important post‐transcriptional regulator of sperm flagellar assembly and motility. KIF6 forms a functional transport module with the RBPs FMRP and FXR1, contributing to the spatiotemporally controlled delivery of mRNAs along the “cytoplasm–connecting piece–flagellum” axis (Figure 9). This process supports the targeted localization and supply of transcripts encoding axonemal assembly proteins (for example, DNALI1 [49]) and motility‐related metabolic enzymes (for example, HK1 [50, 51]). Loss of KIF6 function results in asthenozoospermia and male infertility, not only highlighting its functional necessity during spermiogenesis but also revealing a previously unrecognized mRNA‐targeted transport pathway centered on the KIF6–RBP complex and distinct from the classical protein‐centric IFT pathway. By meeting transcript demands during late spermiogenesis, this pathway may functionally complement canonical IFT, which specializes in the transport of pre‐synthesized proteins, thereby jointly supporting the material requirements for flagellar biogenesis.

This model also helps explain the transcript–protein discordance observed in *Kif6* mutant mice, in which relatively modest changes in steady‐state mRNA abundance were accompanied by much more pronounced defects in flagellar and metabolic‐related proteins. This pattern indicates that the protein‐level defects in *Kif6* mutant sperm cannot be explained solely by changes in steady‐state mRNA abundance. Together with our analyses of KIF6–RBP interactions, reduced flagellar enrichment of selected mRNAs, and decreased puromycin incorporation in *Kif6* mutant sperm flagella, these findings support a post‐transcriptional regulatory model in which KIF6 promotes the delivery and local availability of selected mRNA cargoes associated with flagellar structure and motility.

Although previous studies reported that global deletion of *Kif6* in mice leads to impaired motility and male infertility [34], the molecular mechanisms by which KIF6 regulates sperm development and function remained unexplored. Here, we identify homozygous deleterious *KIF6* variants in men with infertility for the first time and further clarify its role in human spermiogenesis by generating two mouse models that mimic human mutations, thereby providing a model system with closer clinical relevance than global knockout approaches. In the frameshift mutation model, the KIF6 protein is completely absent, abolishing its flagellar localization and disrupting the assembly of the KIF6–FMRP/FXR1 complex (Figure 9B, lower‐middle panel). This defect blocks mRNA transport into the flagellum, reduces the expression of axonemal proteins (such as DNALI1) and metabolic enzymes (such as HK1), and consequently leads to severe axonemal ultrastructural defects, mitochondrial dysfunction, and a completely immotile sperm phenotype. In the missense mutation model, KIF6 protein is stably expressed but displays abnormally enhanced interactions with FMRP/FXR1‐associated messenger ribonucleoprotein (mRNP) granules. This aberrant binding likely traps mRNP granules within the head cytoplasm, reduces their delivery to the flagellum, and ultimately results in markedly impaired sperm motility (Figure 9B, lower‐right panel). Our findings demonstrate that both loss‐of‐function mutations and missense mutations causing complex dysfunction can disrupt KIF6‐mediated mRNA delivery to the flagellum, ultimately impairing sperm motility.

Previous work established KIF6 as a motor for cargo transport localized to motile cilia and capable of processive movement along axonemal microtubules, although its specific cargoes remained unknown [14, 34]. We did not detect interactions between KIF6 and canonical IFT components (Figure 6A). Instead, we identified for the first time that KIF6 transports RBPs such as FMRP and FXR1. Co‐IP and domain mapping analyses confirmed that KIF6 interacts with FMRP and FXR1 through its N‐terminal motor domain and C‐terminal tail domain (Figure S9B,C). Furthermore, we identified a direct intramolecular interaction between the N‐ and C‐terminal domains of KIF6 (Figure S9F). Previous studies have established a conserved regulatory paradigm in which kinesin family members, including KIF17 (kinesin‐2) and CENPE (kinesin‐7), adopt an autoinhibited conformation via intramolecular N–C terminal interactions in the absence of cargo. Cargo binding to the C‐terminal domain relieves autoinhibition and permits microtubule engagement by the N‐terminal motor domain [14, 53, 54, 55]. As a member of the kinesin family, we propose that KIF6 may follow a similar regulatory paradigm, in which N–C intramolecular interactions cooperate with intermolecular KIF6–RBP binding to promote the assembly and transport of functional transport complexes along microtubules; this regulatory model awaits further experimental validation. Although KIF6 and KIF9 are the two members of the kinesin‐9 family, our co‐IP analysis showed that KIF9 did not detectably interact with FMRP or FXR1. This finding suggests that the FMRP/FXR1‐associated mRNA transport mechanism is not a general property of kinesin‐9 motors, but more likely reflects the cargo‐binding specificity of KIF6.

KIF6‐mediated mRNA targeting via RBPs may provide a post‐transcriptional mechanism for adapting protein output to the spatial and temporal constraints of flagellar assembly. Previous studies have detected mRNAs and translation‐associated components within developing flagella [30, 32, 33, 56, 57, 58], suggesting intrinsic translational potential within this organelle. Building on these observations, puromycin labeling in mature epididymal sperm revealed reduced nascent peptide synthesis activity in *Kif6* mutant sperm flagella, supporting impaired flagellar translational activity in mature sperm. However, because this assay was not performed in developing testicular spermatids and does not identify translation from individual KIF6‐targeted transcripts, these data should be interpreted as supportive evidence rather than direct proof of transcript‐specific local translation during flagellar assembly. Thus, our central conclusion remains that KIF6‐RBP complexes mediate mRNA transport during spermiogenesis, while the puromycin data further indicate that *Kif6* mutation is associated with reduced flagellar nascent peptide synthesis in mature sperm. During late spermiogenesis, when global transcriptional activity is largely silenced [59, 60], KIF6‐mediated mRNA targeting may help maintain the local availability of selected transcripts associated with flagellar structure and motility, thereby representing an important post‐transcriptional regulatory pathway at this developmental stage.

Although KIF6 is primarily characterized as a regulator of flagellar assembly, multiple lines of evidence from our study indicate that KIF6 deficiency is also associated with compromised mitochondrial bioenergetic capacity in sperm. In *Kif6* mutant spermatozoa, we observed profound alterations in mitochondrial sheath organization, reduced ATP levels, and coordinated downregulation of proteins involved in oxidative phosphorylation and tricarboxylic acid (TCA) cycle metabolism. In the severe *Kif6* ^M1/M1^ mutant, axonemal disruption occurs together with mitochondrial sheath abnormalities, making it difficult to fully separate direct metabolic effects from secondary consequences of flagellar structural defects. However, several observations suggest that the metabolic abnormalities are not merely secondary to axonemal disorganization. In *Kif6* ^M2/M2^ sperm, axonemal ultrastructure is largely preserved, yet ATP levels and metabolic protein abundance are reduced. Moreover, KIF6–RBP cargo analyses identified transcripts associated with metabolic function, such as *Hk1* and *Qrich2*, whose flagellar enrichment and protein abundance are decreased in mutant sperm. These findings support a model in which KIF6‐mediated mRNA transport contributes to the local availability of selected metabolic transcripts, while mitochondrial sheath defects in the severe frameshift mutant may also be exacerbated by secondary structural disruption.

This study has several inherent limitations. First, only two pathogenic KIF6 variants were identified, and the functional significance of additional KIF6 sites in flagellar assembly remains to be explored. Second, although co‐IP assays and structural modeling suggest that the E474K mutation may enhance KIF6–FMRP/FXR1 interactions, direct biophysical measurements of binding constants by surface plasmon resonance (SPR) or MicroScale Thermophoresis (MST) will be required to validate the predicted changes in binding affinity. Third, while our data support KIF6–RBP‐mediated mRNA targeting to the flagellum, the functional coordination between this pathway and classical IFT‐mediated protein transport remains to be elucidated. Fourth, our functional analyses focused on FMRP and FXR1, leaving other potential KIF6‐interacting RBPs uncharacterized. Fifth, the molecular cues that trigger mRNP release and translational activation within the flagellum remain undefined. Future studies should therefore aim to elucidate the functional significance of additional *KIF6* variants, directly assess local translation in sperm flagella, clarify the interplay between KIF6‐dependent mRNA transport and IFT pathways, comprehensively map the complete RBP interaction network of KIF6, and identify the signals governing mRNP release and translational activation, thereby further refining the molecular regulatory framework of sperm flagellar assembly.

In summary, this study reveals for the first time the critical regulatory role of the KIF6–RBP‐mediated mRNA targeting pathway in sperm flagellar assembly. It expands the traditional protein‐centric paradigm to a molecular framework of coordinated protein‐nucleic acid co‐transport, providing a novel conceptual framework and research perspective for understanding the regulatory network of sperm flagellar biogenesis. This pathway and the canonical IFT protein transport pathway form a spatiotemporally complementary functional system, collectively maintaining the precision and efficiency of flagellar assembly. Evolutionarily, the domains and amino acid sequences of KIF6 are highly conserved from *Chlamydomonas* to humans, highlighting the evolutionary antiquity of kinesin‐mediated mRNA transport mechanisms and providing a conserved paradigm for extending the regulatory model established in this study to cilia/flagella research in other eukaryotes, underscoring the basic scientific value and cross‐species universality of our findings. Clinically, the identification of KIF6 as a novel disease gene for asthenozoospermia, together with the mRNA repertoire targeted by the KIF6–FMRP/FXR1 module, offers new molecular markers for flagellar structural and functional defects. These findings further provide a theoretical foundation and potential strategies for the precise diagnosis and targeted intervention of male infertility.

## 4 Experimental Section

### 4.1 Study Participants

A cohort of 314 Chinese males (aged 22–45 years) diagnosed with asthenozoospermia and 1500 fertile controls (aged 22–40 years) was recruited from the Reproductive and Genetic Hospital of CITIC‐Xiangya (Changsha, China). Asthenozoospermia was defined according to WHO guidelines [61]. All participants had idiopathic infertility, with a normal karyotype (46, XY) and absence of Y‐chromosome microdeletions. Clinical evaluation excluded other potential causes of infertility, including genital tract infection, testicular inflammation, iatrogenic injury, and drug exposure. Physical examination revealed no abnormalities in external genitalia. The study was approved by the Ethics Committee of the Reproductive and Genetic Hospital of CITIC‐Xiangya (Approval No. LL‐SC‐2019‐034), and written informed consent was obtained from all participants prior to enrollment.

### 4.2 Whole‐Exome Sequencing and Sanger Sequencing

Whole‐exome sequencing (WES) and Sanger sequencing were performed as previously described [62]. Genomic DNA was extracted from peripheral blood lymphocytes using the QIAamp DNA Blood Midi Kit (Qiagen, Germany, 51106) following the manufacturer's protocol. Exome capture was achieved with the Agilent SureSelect Human All Exon V6 Kit (Agilent Technologies, Santa Clara, CA, USA), followed by next‐generation sequencing on the Illumina HiSeq 2000 (Illumina, San Diego, CA, USA). The raw sequencing data were aligned to the human genome reference assembly (GRCh37/hg19) using Burrows‐Wheeler Aligner (BWA) software. Annotation was performed using ANNOVAR to retrieve variant loci, genotype, zygosity, population allele frequencies (1000 Genomes Project, Exome Aggregation Consortium, gnomAD), and pathogenicity predictions (SIFT, Polyphen‐2, MutationTaster, CADD). Variants were filtered based on the following criteria: minor allele frequency (MAF) < 0.01 in public control databases, non‐synonymous or frameshift annotations, and predicted deleterious by at least two of the four pathogenicity predictors. Identified *KIF6 *variants (c.1325_1326del, p.T442fs; c.1420G>A, p.E474K) were validated by Sanger sequencing using primers listed in Table S5.

### 4.3 Semen Analysis

Semen analyses followed WHO guidelines [61, 63, 64]. Semen samples from patients were collected via masturbation following 2–7 days of sexual abstinence, then liquefied at 37°C for 30 min prior to analysis. Sperm motility parameters were assessed using the Computer Assisted Sperm Analysis (CASA) system. Sperm morphological analysis was carried out by H&E staining.

For mouse sperm quality assessment, spermatozoa were extracted from the cauda epididymis and vas deferens, incubated at 37°C for 30 min, and counted with a hematocytometer. Mouse sperm morphology was analyzed by H&E staining. Sperm motility was assessed using the CASA system.

### 4.4 Animal Models

The *Kif6* ^M1/M1^ and *Kif6* ^M2/M2^ mouse models were generated using CRISPR/Cas9 technology from Cyagen Biosciences (Guangzhou, China). Briefly, for *Kif6* ^M1/M1^ mice, Cas9 mRNA and in vitro transcribed sgRNAs were co‐injected into C57BL/6 mouse zygotes, followed by transplantation into pseudopregnant surrogate females at the two‐cell stage. For *Kif6* ^M2/M2^ mice, Cas9 mRNA, sgRNAs, and a donor vector were co‐injected into zygotes. The donor vector was constructed using homology arms generated by PCR, with the BAC clone RP23‐351P1 from the C57BL/6J library serving as the template. F0 founder mice were identified through PCR and subsequent sequence analysis. These founders were bred to WT mice to assess germline transmission and to generate F1 animals. Confirmed F1 heterozygous mutants were intercrossed to produce homozygous *Kif6* ^M1/M1^ and *Kif6* ^M2/M2^ mice for use in this study. The genotyping primers are detailed in Table S6.

All mice were housed under standard conditions at Central South University Animal Services (Changsha, China). All animal experimental procedures adhered to the protocols set forth by the Institutional Animal Care and Use Committees of Central South University (CSU‐2022‐0001‐0261).

### 4.5 RNA Isolation and Quantitative Real‐Time PCR

Total RNA was extracted from testicular tissues using TRIzol reagent (15596018CN, Thermo Fisher Scientific). RNA quality was assessed with a NanoDrop One spectrophotometer (A260/A280 = 1.8–2.0). cDNA was synthesized from 1000 ng total RNA using the PrimeScript RT Reagent Kit (TaKaRa, RR047A) with gDNA Eraser in a 20 µL reaction. Quantitative RT‐PCR was performed with SYBR qPCR Master Mix (Vazyme, Q711‐02) on a Roche Lightcycler 480 system using primers listed in Table S7. Relative expression was calculated by the 2^−ΔΔCt^ method normalized to Gapdh.

### 4.6 RNA Immunoprecipitation

RNA immunoprecipitation (RIP) assay was conducted with minor modifications to the previously described method [65]. Briefly, testicular tissues from WT mice were homogenized in lysis buffer (100 mM KCl, 5 mM MgCl_2_, 10 mM HEPES, 0.5% NP‐40) supplemented with RNase inhibitor (TaKaRa) and protease inhibitor cocktail (Ncmbio) on ice. Protein A/G Magnetic Beads (MCE, HY‐K0202) were washed with lysis buffer and incubated overnight at 4°C with 5 µg antibody (anti‐FMRP, anti‐FXR1, anti‐KIF6) or control IgG (Beyotime, A7016). Lysate supernatant was added to antibody‐coated beads and incubated with rotation at 4°C for ≥4 h. Beads were washed twice with lysis buffer and three times with wash buffer (50 mM Tris‐HCl pH 7.5, 500 mM NaCl, 5 mM EDTA, 0.1% Triton X‐100) containing protease inhibitor and RNase inhibitor. Co‐precipitated RNA was eluted by incubating beads in 100 µL proteinase K buffer (wash buffer with 2.5 µL 20 mg/mL Proteinase K [Coolaber, SL20741] and 1 µL 10% SDS) at 55°C for 30 min. Supernatant was collected, and RNA was extracted with RNAiso Plus (Takara, 9108). RNA purity was verified, followed by reverse transcription and qRT‐PCR or RNA‐seq analysis. FMRP target genes were identified with edgeR (FDR< 1, fold change ≥1.5); genes with log_2_ fold change >1 were selected for further study. Primers are listed in Table S7.

### 4.7 RNA‐Seq Analysis

Testes were dissected from adult (two‐month‐old) *Kif6* ^+/+^ and *Kif6* ^M1/M1^ mice for bulk RNA‐seq analysis, following the previously described protocol [65]. Differential expression analysis used DESeq2 (v1.4.5), with differentially expressed genes (DEGs) defined as *p*< 0.05 and |log_2_ fold change |≥1.

### 4.8 Proteomic Analysis of Testicular Tissues or Sperm

Proteins were extracted from testicular tissues or sperm of adult *Kif6* ^+/+^ and *Kif6* ^M1/M1^ mice using a Tendon/Ligament Total Protein Extraction Kit (Invent Biotechnologies, SA‐04‐TD). Protein concentration was measured with a BCA assay (Thermo Fisher Scientific, 23225). Digestion followed a modified filter‐aided sample preparation (FASP) protocol: [66] equal protein amounts were loaded onto 10K MWCO centrifugal filters (Sartorius, VN01H02), washed with 8 M urea in 50 mM triethylammonium bicarbonate (TEAB), reduced with 100 mM DTT (Sigma‐Aldrich, D9779) at 56°C for 1 h, alkylated with 100 mM iodoacetamide (Sigma–Aldrich, I1149) for 40 min at room temperature in the dark, and digested with trypsin (Hualishi Scientific, HLS TRY001C; 1:50 enzyme‐to‐substrate ratio) at 37°C for 16 h. Peptides were collected by centrifugation (15 000 × *g*, 15 min).

Mass spectrometry (MS) analysis was performed with our optimized protocol: [67] peptides were analyzed on a Thermo Fisher Orbitrap Eclipse Tribrid mass spectrometer equipped with a FAIMS Pro Interface. Peptides were desalted on a 2 cm PepMap trap column and separated on a 25 cm PepMap analytical column with a 3%–38% buffer B (80% acetonitrile, 0.1% formic acid) gradient over 102 min. FAIMS used compensation voltages of −35, −45, and −65 V. MS1 spectra were acquired in the Orbitrap (resolution 60k; mass range 350–1500 m/z) with 20‐s dynamic exclusion. MS2 spectra were collected in the linear ion trap using HCD (collision energy 35%).

Raw data were processed with Proteome Discoverer (v2.5). MS/MS spectra were searched against the UniProt mouse database (downloaded 24 June 2022; 55 286 entries) with fixed carbamidomethylation of cysteine, variable oxidation of methionine and N‐terminal acetylation, precursor tolerance 10 ppm, fragment tolerance 0.6 Da, maximum two missed cleavages, and peptide length 6–144 amino acids. Protein identification FDR was set to <0.01. Protein intensities were normalized with vsn, and missing values were imputed using the R package DEPs (v1.20.0). Differential expression analysis used limma (v3.54.2); significant proteins were defined as *p*< 0.05 and |log_2_ fold change| ≥1. Functional enrichment was performed with Metascape (https://www.metascape.org).

### 4.9 Proteomic Analysis of Sperm Flagella

#### 4.9.1 Flagella Isolation

Sperm flagella were enriched from mouse cauda epididymides as described with minor modifications [68]. Cauda epididymides from seven adult male mice were incubated in physiological saline at 37°C for 30 min to release sperm. The sperm suspension was centrifuged (800 × *g*, 5 min, 4°C), and the pellet was washed three times with ice‐cold 75 mM NaCl to remove somatic cells. The washed pellet was resuspended in 1 mL TBS and sonicated using an ultrasonic cell disruptor (Scientz‐IID, Ningbo Scientz Biotechnology) at 60 W for two cycles (1 s on, 1 s off per cycle; 10 s total) to separate heads from tails, with separation confirmed by light microscopy. After centrifugation (15 000 × *g*, 5 min, 4°C), the pellet was resuspended in 1 mL of 80% Percoll (GE Healthcare) in TBS and centrifuged again (15 000 × *g*, 15 min, 4°C). The upper flagella‐enriched fraction was collected, diluted with 6–8 volumes of TBS, and pelleted by centrifugation (15 000 × *g*, 5 min, 4°C). Flagellar purity was assessed by light microscopy.

#### 4.9.2 Sample Processing

Flagellar‐enriched samples were lysed in buffer containing 0.01% n‐dodecyl‐β‐D‐maltoside (DDM), protease inhibitor cocktail (1%), and 100 mM TEAB (pH 8.5), followed by ultrasonication for 10 cycles (30 s on / 30 s off) at 4°C. Lysates were reduced with 100 mM DTT for 40 min at 75°C and alkylated with 100 mM IAA for 30 min at room temperature in the dark. Proteins were digested with trypsin (Hualishi Scientific, HLS TRY001C) at a final concentration of 10 ng/µL and incubated at 37°C for 4 h. An equivalent amount of trypsin was added, and digestion was continued overnight at 37°C. The final lysates were desalted by a homemade C18 SPE column and vacuum‐dried for the proteomic analysis.

#### 4.9.3 Contamination Assessment

Nuclear proteins (Lamins A/C, PRM1, PRM2) were used as markers to evaluate sperm‐head contamination.

### 4.10 Mass Spectrometry Analysis of Pull‐Down Proteins

In vivo immunoprecipitation assays were conducted to identify KIF6‐interacting proteins. Testicular tissues from adult *Kif6* ^+/+^ mice were washed twice with PBS and lysed using NP‐40 (Beyotime, P0013F) containing a Protease Inhibitor Cocktail. The lysate was incubated overnight at 4°C with rotation after the addition of either an anti‐KIF6 antibody (1:100, Proteintech, 17290‐I‐AP) or an IgG antibody. Subsequently, the complexes were incubated with Protein A/G Magnetic Beads (MCE, HY‐K0202) for 2 h at 4°C. Following three washes with lysis buffer and five washes with PBS, all samples were subjected to MS analysis.

### 4.11 Untargeted Sperm Metabolomics Analysis

Sperm were isolated from the cauda epididymis of adult WT and mutant mice (n = 4 per genotype) as detailed in the Semen analysis section. Sperm pellets were resuspended in 250 µL ice‐cold methanol spiked with internal standards (0.50 µg/mL FFA (C16:0‐d3), 0.69 µg/mL PE (30:0) and 0.94 µg/mL LPC (19:0)). The mixture was vortexed for 5 min, incubated at ‐20°C for 10 min, and centrifuged (14 000 × *g*, 4°C, 15 min). A 100 µL aliquot of the supernatant was collected, lyophilized using a low‐temperature vacuum centrifugal concentrator (Labconco Corporation, USA), and stored at −80°C until analysis.

Quality control (QC) samples were obtained from pooled metabolic extracts and prepared as real samples. Metabolite profiling was performed using a UHPLC‐Orbitrap Exploris MS system comprising a Vanquish Flex UHPLC system (Thermo Scientific, USA) coupled to an Orbitrap Exploris 120 mass spectrometer (Thermo Scientific, USA). Separation was achieved on a Hypersil GOLDTM VANQUISHTM C18 column (150 × 2.1 mm^2^, 1.9 µm; Thermo Scientific, USA) with mobile phase: (A) ultrapure water containing 5 mM ammonium formate (NH_4_FA) and 0.05% formic acid and (B) acetonitrile; A gradient elution program was applied as follows: 2% B (0–2 min), linear increase to 70% B (2–7 min), 70%–90% B (7–14 min), 90%–100% B (14–16 min), isocratic at 100% B (16–20 min), rapid reduction to 2% B (20–20.1 min), and equilibration at 2% B (20.1–25 min). The UHPLC system was operated at a flow rate of 0.3 mL/min, column temperature of 40°C, and injection volume of 5 µL. Mass spectrometry data were acquired in full‐scan and data‐dependent MS2 (ddMS2) modes using electrospray ionization (ESI) in both positive and negative polarities. Key parameters included: spray voltage (± 5.5 kV), capillary temperature (325°C), vaporizer temperature (400°C), sheath gas flow (50 arbitrary units), auxiliary gas flow (15 arbitrary units), and normalized collision energy (NCE) of 20, 35, and 50. Full‐scan resolution was set to 140 000 (at m/z 200), and ddMS2 resolution to 35 000, with a scan range of m/z 80–1000. The TopN setting (10 most abundant ions per cycle) was applied for fragmentation.

Metabolomics data were processed using Compound Discoverer 3.3 software (Thermo Fisher Scientific) for peak alignment, detection, filtering, and metabolite annotation. Routine parameters were used for peak processing, and poorly repeatable features (detection rate ≤ 50% and CV ≥ 30% in QC samples) were filtered out. Metabolites were putatively annotated using the ChemSpider database by comparing theoretical and detected MS data and also the mzCloud database by matching the reference and detected MS/MS spectrum. Finally, QC‐based correction was performed using the statTarget R package. Differential metabolites were identified using the limma package and subjected to KEGG pathway enrichment analysis via MetaboAnalyst 6.0 (http://www.metaboanalyst.ca).

### 4.12 Proteomics and Metabolomics Joint‐Pathway Analysis

We utilized MetaboAnalyst 6.0 (https://www.metaboanalyst.ca/) for joint pathway analysis of proteomics and metabolomics data, and chose the all‐pathway enrichment for this analysis.

### 4.13 Cell Culture and Plasmid Construction

HEK293T cells (Procell) were authenticated by STR profiling and cultured in DMEM (Gibco) supplemented with 10% FBS (Sigma‐Aldrich), penicillin (100 U/mL), and streptomycin (100 µg/mL) at 37°C in 5% CO_2_. Plasmids encoding human *KIF6* (NM_145027.6; pCMV3‐FLAG‐KIF6‐t1), mouse *Kif6* (NM_177052.3; pCMV3‐Flag‐mKif6), human *FXR1* (NM_005087.2; pCMV3‐FXR1‐Myc), mouse Fmr1 (NM_008031.4; pCMV3‐mFmr1‐t1‐Myc), and human *KIF9* (NM_182902.3; pCMV3‐KIF9‐HA) were from Sino Biological (Beijing). pCMV‐EGFP‐FMR1‐Neo (P47185) was from Miaolingbio (Wuhan). *Fxr1* (NM_001113188) and *Fxr2* (NM_011814) were amplified from mouse testis cDNA and cloned into Myc‐ or Flag‐tagged vectors using the ClonExpress II One Step Cloning Kit (Vazyme, C112‐02). Tag insertion (FLAG, HA, Myc) was performed with the same kit. Mutations and truncations were introduced using the Mut Express II Fast Mutagenesis Kit V2 (Vazyme, C214‐01) or PCR‐based cloning. All plasmids were transfected into HEK293T cells using the Neofect DNA transfection reagent (Neofect Biotech, TF201201) according to the manufacturer's protocol.

### 4.14 Western Blotting and Co‐Immunoprecipitation

Cells or testicular tissues were lysed in RIPA buffer (Beyotime, P0013B) with a protease inhibitor cocktail. Lysates were heated with loading buffer at 100°C for 10 min, separated by SDS‐PAGE, and transferred to PVDF membranes (Millipore). Membranes were blocked with 5% non‐fat milk in TBST (2 h, room temperature), incubated overnight at 4°C with primary antibodies (Table S8), and probed with secondary antibodies (2 h, room temperature). Signals were quantified with ImageJ.

For Co‐IP, proteins were extracted with NP‐40 lysis buffer containing protease inhibitors and 1 mM PMSF. Lysates were incubated overnight at 4°C with specific antibodies, then with Protein A/G Magnetic Beads for 2 h at 4°C. Beads were washed three times with NP‐40 buffer (with PMSF) and twice with PBS. Bound proteins were eluted in denaturing SDS buffer (95°C, 10 min) and analyzed by Western blotting.

### 4.15 Immunofluorescence

Spermatozoa/spermatid smears were prepared on poly‐L‐lysine‐coated slides, fixed with 4% PFA (15 min, room temperature), permeabilized with 0.5% Triton X‐100 (1 h, room temperature), and blocked with 5% goat serum (1 h, room temperature). Samples were incubated overnight at 4°C with primary antibodies against KIF6, FMRP, FXR1, or α‐Tubulin (Table S8), followed by Alexa Fluor 488‐ or 594‐conjugated secondary antibodies (2 h, 37°C). Nuclei were stained with DAPI. Images were captured on an Olympus BX53F microscope with consistent settings. Fluorescence intensity was quantified with ImageJ.

### 4.16 Spermatocyte Spreads

Spermatocyte spreads were made as previously described [65]. Briefly, testes from adult control and mutant mice were dissected, decapsulated, and seminiferous tubules were gently dispersed in phosphate‐buffered saline (PBS). Tubules were incubated in hypotonic buffer for 30 min to release spermatocytes, followed by gentle mechanical dissociation. The resulting cell suspension was spread onto glass slides containing fixative (1% PFA, 0.15% Triton X‐100) and incubated in a humid chamber overnight at room temperature. Slides were then air‐dried, washed with PBST, and processed for immunofluorescence.

### 4.17 Testicular Squash Preparation

Testes were dissected from mice and placed in ice‐cold PBS. The tunica albuginea was carefully removed with fine forceps, and seminiferous tubules were gently separated using a pipette. Tubules were fixed in 2% PFA at RT for 10 min with gentle shaking. After fixation, 2–3 tubules were selected and placed on a glass slide, covered with a coverslip, and evenly pressed by hand to spread the tubules into a thin layer. Samples were snap‐frozen in liquid nitrogen for 5 min, and the coverslip was quickly removed with a razor blade. Samples were stored at −80°C for subsequent immunofluorescence.

### 4.18 Preparation of Single Germ Cell Suspensions

#### 4.18.1 Isolation of Cytoplasmic‐Containing Spermatids

After removing the tunica albuginea, testes were minced into small pieces and digested with 1 mg/mL collagenase IV (Gibco; 17104‐019) in DMEM (Gibco) in a 37°C water bath for 3–5 min with gentle shaking. The collagenase solution was removed by centrifugation at 500 × *g* for 5 min at 4°C, and the tissue pellet was resuspended in 0.25% trypsin (Gibco; 25200056) containing 10 µg/mL DNase I (Solarbio; D8071) and incubated at 37°C for 5–8 min, with repeated pipetting every 2 min until no tissue fragments remained. Digestion was stopped by adding an equal volume of DMEM supplemented with 10% FBS. The cell suspension was filtered through a 70 µm cell strainer (BD Biosciences) to remove debris, and centrifuged at 500 × *g* for 5 min at 4°C. The pellet was resuspended in DMEM and centrifuged again at 500 × *g* for 5 min. Finally, cells were resuspended in normal saline and gently pipetted approximately 50 times to disperse cell clumps. The resulting suspension was used to prepare smears for subsequent Immuno‐FISH.

#### 4.18.2 Isolation of Flagellated Spermatids From Testes

Separation of tails from testicular spermatids was performed with slight modifications based on previously published protocols [32, 68 ]. Testes from sexually mature mice (12–15 weeks old) were dissected, the tunica albuginea was removed, and tissues were minced in 1 mL modified Whitten's media (22 mM HEPES、1.2 mM MgCl_2_、100 mM NaCl、4.7 mM KCl、1.0 mM pyruvate、5.5 mM glucose、4.8 mM lactate, pH 7.3) supplemented with 1 mM PMSF and 1 µL RNase inhibitor (TaKaRa) in a 35 mm culture dish. The tissue suspension was transferred to a 1.5 mL centrifuge tube and centrifuged briefly (100 × *g* for 1 min) to remove large tissue fragments, after which the supernatant was transferred to a new 1.5 mL centrifuge tube. After centrifugation at 10 000 × *g* for 5 min at 4°C, the pellet was collected and washed three times with cold 75 mM NaCl (centrifuged at 10 000 × *g* for 5 min each time). The pellet was resuspended in 30% Percoll solution (GE Healthcare) diluted in TBS, and centrifuged at 15 000 × *g* for 20 min at 4°C. The middle filamentous layer (enriched in flagellated spermatids) was carefully collected with a pipette, resuspended in PBS, and centrifuged at 10 000 × *g* for 5 min to obtain the final cell pellet. The pellet was used to prepare smears for subsequent Immuno‐FISH.

### 4.19 Immuno‐FISH

RNA fluorescence in situ hybridization (RNA‐FISH) was performed per the manufacturer's instructions (Gene Pharma, Shanghai, China) with minor modifications. Briefly, cells on slides were fixed with 4% PFA for 15 min at RT, washed twice with PBS (5 min each), and permeabilized with 0.5% Triton X‐100 in PBS for 15 min at RT. Following a PBS wash (5 min), slides were incubated with 2× SSC buffer at 37°C for 30 min to rehydrate. Pre‐designed Cys‐modified probes targeting *Dnali1* (5'‐ TGAAG+TCAATCAGGATGT+TACGG‐3', T+TCAACATC+TACAGCCTGTCCTC, CCTCGCTTCT+TAATGGCTT+TATTC) and *Hk1* (5'‐GGAGA+TGCACCACATCCAT+TC‐3', GGTGCGATAATGCCT+TCCAGT, CT+TCTGCCTGTT+TGGTAGGATCTGG) were obtained from Gene Pharma. A probe mixture targeting *Dnali1* and *Hk1* (8 µM each) was prepared in hybridization buffer, denatured at 73°C for 5 min, and immediately placed on ice for 5 min. The denatured probe mixture was applied to the smears, covered with a coverslip, and incubated overnight at 37°C in a humidified hybridization chamber.

The following day, the coverslip was removed, and slides were sequentially washed with pre‐warmed (42°C) 4×SSC containing 0.1% Tween 20 (5 min), 2× SSC (5 min), and 1× SSC (5 min) in a water bath. After air‐drying at RT for 10 min, slides were washed with PBS (5 min) and blocked with 5% BSA in PBS for 1 h at RT. Standard immunofluorescence staining was then performed as described above, using primary antibodies against KIF6, FMRP, or FXR1 and corresponding secondary antibodies. Nuclei were counterstained with DAPI. Images were captured using an Olympus BX53F fluorescence microscope equipped with a 60× oil immersion objective. Colocalization analysis was performed using the Coloc 2 plugin in ImageJ software (1.54p), with additional plot profile analysis conducted for quantitative assessment of signal distribution. Quantitative analysis of the fluorescence intensity of HK1/DNALI1 proteins and *Hk1*/*Dnali1* mRNAs was performed using ImageJ software (v1.54p) with automated region‐of‐interest (ROI) analysis.

### 4.20 Testicular Sperm Tail Enrichment

Testes from sexually mature mice were dissected, decapsulated, and minced in 1 mL modified Whitten's media supplemented with 1 mM PMSF and 1 µL RNase inhibitor (TaKaRa) in a 35 mm culture dish. The tissue suspension was centrifuged at 100 × *g* for 1 min to remove large debris, and the supernatant was subsequently centrifuged at 10 000 × *g* for 5 min at 4°C to pellet testicular sperm. The pellet was washed three times with ice‐cold 75 mM NaCl. The washed pellet was resuspended in 500 µL 30% Percoll solution (GE Healthcare) diluted in TBS, transferred to a 1.5 mL centrifugal tube, and centrifuged at 15 000 × *g* for 15 min at 4°C. The upper layer was carefully removed, and the remaining fraction was resuspended in 1 mL PBS, centrifuged at 10 000 × *g* for 5 min to collect the pellet. The pellet was resuspended in 200 µL PBS and sonicated using an ultrasonic cell disruptor (Scientz‐IID, Ningbo Scientz Biotechnology, China) at 50 W for 6 s (on 1s, off 1s) for 2 cycles. The sonicated suspension was examined under a light microscope to confirm separation of testicular sperm heads and tails. It was then centrifuged at 15 000 × *g* for 5 min at 4°C to collect the pellet, which was resuspended in 500 µL of 80% Percoll (GE Healthcare) diluted in TBS and centrifuged again at 15 000 × *g* for 15 min at 4°C. The upper supernatant, containing pure testicular sperm tails, was collected, transferred to a new 1.5 mL tube, and mixed with 6 volumes of PBS. This mixture was centrifuged at 10 000 × *g* for 5 min at 4°C to harvest the testicular sperm tails. The resulting pellet was snap‐frozen in liquid nitrogen and stored at −80°C for subsequent RT–qPCR analysis.

### 4.21 Puromycin Labeling Assay

Puromycin nascent chain labeling was performed as previously described with minor modifications [58]. Briefly, cauda epididymides and vas deferens were dissected from adult WT, *Kif6* ^M1/M1^, and *Kif6* ^M2/M2^ mice and incubated in pre‐equilibrated Human Tubal Fluid (HTF) medium at 37°C with 5% CO_2_ for 1 h to allow sperm release; sperm from poorly released samples were gently collected by mechanical extrusion. The sperm suspension was carefully collected and divided into three groups: no‐puromycin control, puromycin labeling, and cycloheximide (CHX)‐treated control. Sperm were incubated with 10 µg/mL puromycin for 10 min at 37°C, whereas CHX‐treated samples were preincubated with 100 µg/mL CHX for 30 min before puromycin addition. Reactions were terminated by adding an equal volume of ice‐cold PBS containing CHX. After centrifugation and washing with ice‐cold PBS, sperm were attached to poly‐L‐lysine‐coated slides, fixed with 4% paraformaldehyde, and subjected to antigen retrieval in modified sodium citrate buffer. Samples were then permeabilized with 0.5% Triton X‐100, blocked with 5% BSA, and immunostained with anti‐puromycin and anti‐α‐tubulin antibodies, followed by fluorescent secondary antibodies and DAPI‐containing antifade mounting medium. Images were acquired by fluorescence microscopy.

### 4.22 Molecular Docking

Docking between KIF6^386–566^ and FMRP^1–216^/FXR1^1–216^ was performed with ZDOCK (v3.0.2) [69]. 3D structures of KIF6 (AF‐Q6ZMV9‐F1‐KIF6), FMRP (AF‐Q06787‐F1‐FMR1), and FXR1 (AF‐P51114‐F1‐FXR1) were downloaded from the AlphaFold Database (https://alphafold.ebi.ac.uk/). The 3D structure of mutant KIF6 (p.E474K) was constructed using AlphaFold 3 (https://alphafoldserver.com/) with the wild‐type structure as a template. All structures were prepared in PyMOL 3.0.3. Docking was performed with default parameters as described in the ZDOCK server. The top 10 docking poses were generated for each complex. Based on the docking score, the optimal docking pose of top‐ranked complexes was subject to visual analysis using the software PyMoL 3.0.3. MM‐GBSA binding free energy was calculated by HawkDock [70].

### 4.23 Molecular Dynamics Simulations

Molecular dynamics (MD) simulations were performed on WT and E474K mutant KIF6^3^ ^8^ ^6^^−^^5^ ^6^ ^6^–FMRP^1^^−^^2^ ^1^ ^6^/FXR1^1^^−^^2^ ^1^ ^6^ complexes using Desmond/Maestro (v2022.1, Schrödinger Inc., New York, NY, USA) [71]. TIP3P water molecules were added to the systems, which were then neutralized by 0.15 M NaCl solution. After minimization and relaxation of the system, the production simulation was performed for 100 ns in an isothermal‐isobaric ensemble at 300 K and 1 bar. Trajectory coordinates were recorded every 100 ps. MD analysis was performed using the Simulation Interaction Diagram from Desmond.

### 4.24 Histology and TUNEL Assay

For histological analysis, fresh testes and cauda epididymis samples were fixed in 4% PFA for 24–48 h, followed by paraffin embedding, sectioning, and processing. Sections were stained with H&E or Periodic Acid‐Schiff (PAS) Staining (G1281, Solarbio).

TUNEL assay was performed to detect apoptotic germ cells using the DeadEnd Fluorometric TUNEL System kit (Promega, G3250) according to the manufacturer's instructions. Images were captured with an Olympus BX53F fluorescence microscope (Olympus, Tokyo, Japan).

### 4.25 High‐Speed Video Microscopy Analysis

Ciliary beat activity in mouse tracheobronchial tissues was examined by high‐speed video microscopy. Mice were euthanized according to approved animal protocols, and the trachea was carefully exposed without mechanical stretching or disruption. The trachea and proximal bronchi containing the tracheal bifurcation were isolated after removal of the esophagus and surrounding connective tissues. The airway segment was excised by cutting between the bifurcation and the lungs and at approximately one third of the tracheal length above the bifurcation. The isolated tissues were immediately transferred into prewarmed cilia culture medium and cut into approximately 1 mm × 1 mm fragments. Tissue fragments were maintained at 37°C and examined under a microscope. Videos were captured at 500 frames per second using cellSens Dimension software (Olympus). Multiple fields were recorded from each mouse. Ciliary beat frequency was quantified from the acquired videos using CiliarMove software, as previously described [72]. For statistical analysis, values from multiple fields were averaged for each mouse, and each mouse was treated as one biological replicate.

### 4.26 Scanning and Transmission Electron Microscopy

For scanning electron microscopy (SEM), murine bronchial samples were fixed in 2.5% phosphate‐buffered glutaraldehyde for 2 h at 4°C and then mounted onto poly‐L‐lysine‐coated coverslips. The samples were dehydrated through a chilled graded ethanol series of 50%, 70%, 95%, and 100%, followed by critical‐point drying using a Quorum K850 Critical Point Dryer. The samples were then examined using an S‐3400N scanning electron microscope (Hitachi, Japan).

For transmission electron microscopy (TEM), sperm and bronchial samples were fixed in 2.5% glutaraldehyde and post‐fixed with 1.0% osmium tetroxide. After dehydration through graded ethanol concentrations, the samples were embedded in a resin mixture containing Epon812, dodecenylsuccinic anhydride, methylnadic anhydride, and dimethylaminomethyl phenol. Ultrathin sections of 70–90 nm were prepared and stained with uranyl acetate and lead citrate. Images were acquired using an HT7700 Hitachi electron microscope (Hitachi) equipped with a MegaView III digital camera (Munster).

### 4.27 Measurement of Sperm ATP

For ATP measurement, the cauda epididymis sperm suspension was centrifuged at 500 × *g* for 5 min. The pellet was then resuspended in 100–150 µL ATP‐releasing agent and incubated at 4°C for 10 min. Following a second centrifugation at 12 000 × *g* for 5 min, the supernatant was collected and used to determine ATP levels using an enhanced ATP assay kit (Beyotime, S0027) in a luminometer (Synergy LX; BioTek, USA).

### 4.28 Statistical Analysis

Statistical analysis was performed using GraphPad Prism 9.0 software. Statistical analysis was performed using Student's *t*‐test or ANOVA. Data are presented as mean ± standard error of the mean (SEM) unless otherwise stated.

## Author Contributions

Yue‐Qiu Tan and Chaofeng Tu designed the experiments. Chunbo Xie, Sibing Yi, and Xinle Lin performed the experiments. Weili Wang participated in editing the final manuscript. Shen Zhang participated in proteomics and metabolomics sequencing. Sibing Yi, Lanlan Meng, and Chen Tan processed and analyzed the sequencing data. Yong Li, Chunjia Wei, Yanyan Yu, Yaoqiong Liang, Huan Zhang, Wenbin He, Juan Du, Qianjun Zhang, and Guangxiu Lu provided various technical assistance. Ge Lin participated in project supervision. Liang Hu contributed to data interpretation. Chunbo Xie, Chaofeng Tu, and Yue‐Qiu Tan wrote the paper.

## Conflicts of Interest

The authors declare no conflicts of interests.

## Supporting information




**Supporting File 1**: advs76263‐sup‐0001‐SuppMat.docx.

**Supporting File 2**: advs76263‐sup‐0002‐DataFileS1.xlsx.

**Supporting File 3**: advs76263‐sup‐0003‐DataFileS2.xlsx.

**Supporting File 4**: advs76263‐sup‐0004‐DataFileS3.xlsx.

**Supporting File 5**: advs76263‐sup‐0005‐DataFileS4.xlsx.

**Supporting File 6**: advs76263‐sup‐0006‐DataFileS5.xlsx.

**Supporting File 7**: advs76263‐sup‐0007‐DataFileS6.xlsx.

**Supporting File 8**: advs76263‐sup‐0008‐DataFileS7.xlsx.

**Supporting File 9**: advs76263‐sup‐0009‐DataFileS8.xlsx.

**Supporting File 10**: advs76263‐sup‐0010‐DataFileS9.xlsx.

## Data Availability

The raw whole‐exome sequencing data from the two patients carrying *KIF6* variants reported in this study have been deposited in the Genome Sequence Archive for Human (GSA‐Human) at the National Genomics Data Center, China National Center for Bioinformation/Beijing Institute of Genomics, Chinese Academy of Sciences, under accession number HRA018594, associated with BioProject PRJCA064672. These data are available under restricted access because individual‐level human genomic sequencing data are protected owing to patient privacy and the Regulations on the Management of Human Genetic Resources of China. Access to the raw data can be requested through the GSA‐Human system and may be authorized by the Data Access Committee for research and non‐commercial purposes only. The RNA‐seq datasets generated and used in this study are available in the Zenodo repository at https://doi.org/10.5281/zenodo.20270942. The RIP‐seq data generated in this study have been deposited in the Gene Expression Omnibus (GEO) database under accession number GSE331445. The mass spectrometry proteomics data generated in this study have been deposited in the ProteomeXchange Consortium via the iProX partner repository under accession code PXD078549. Other data supporting the findings of this study are available within the article and its supplementary information files. Additional analysis scripts and relevant materials are available from the corresponding authors upon reasonable request.
