# Supplementary material for: The KIF6‐RBP Complex Orchestrates mRNA Transport Required for Sperm Flagellar Assembly
Source: Adv Sci (Weinh). 2026 Jun 25:e76263. Online ahead of print. doi: 10.1002/advs.76263 (PMC13335463; doi:10.1002/advs.76263)
Supplement: Supplementary file 1 — Supporting File 1: advs76263‐sup‐0001‐SuppMat.docx. [file ADVS-9999-e76263-s001.docx]

Supplementary Materials for

**The KIF6-RBP Complex Orchestrates mRNA Transport Required for Sperm Flagellar Assembly**

Chunbo Xie *et al.*

*Corresponding author. Email: tanyueqiu@csu.edu.cn (T.Y.); 222041@csu.edu.cn (T.C.)

**This PDF file includes:**

Figs. S1 to S14

Tables S1 to S8

**Other Supplementary Materials for this manuscript include the following:**

Data S1 to S9

**
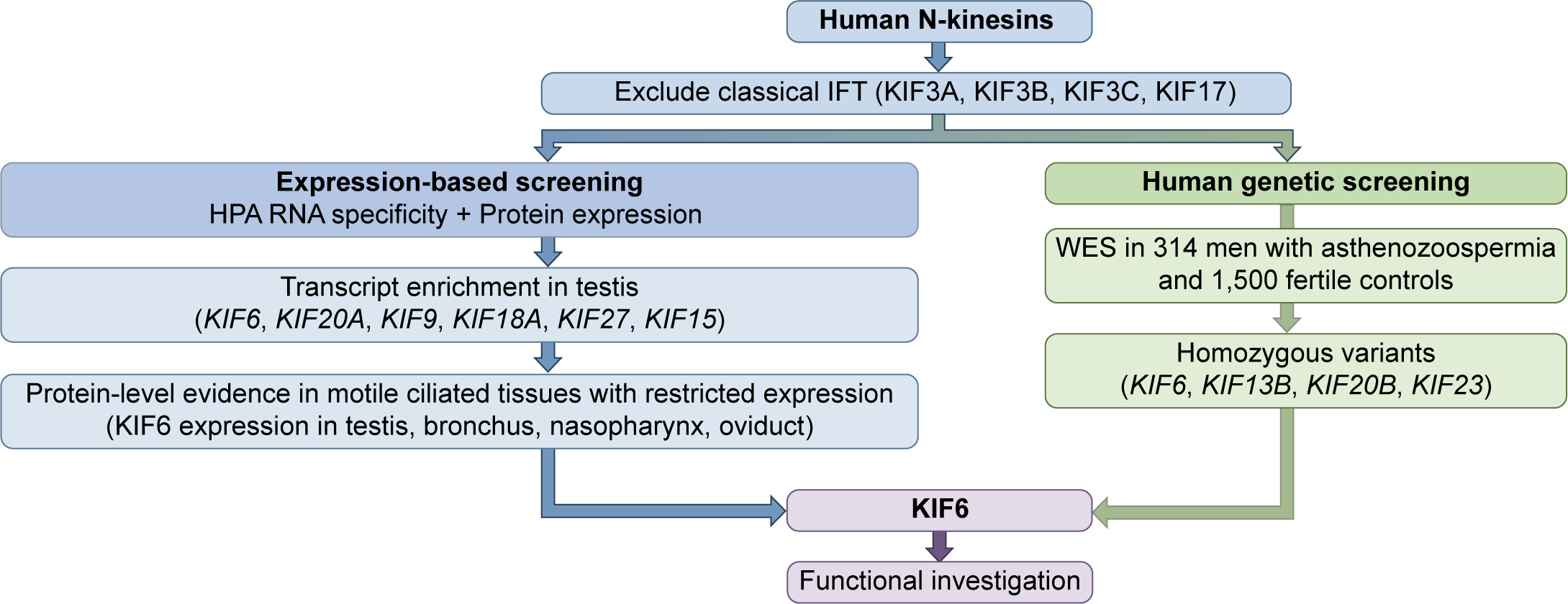
**

**Figure S1. KIF6 as a candidate for human sperm flagellar assembly.** Flowchart illustrating the strategy used to identify kinesin genes potentially involved in sperm flagellar assembly.


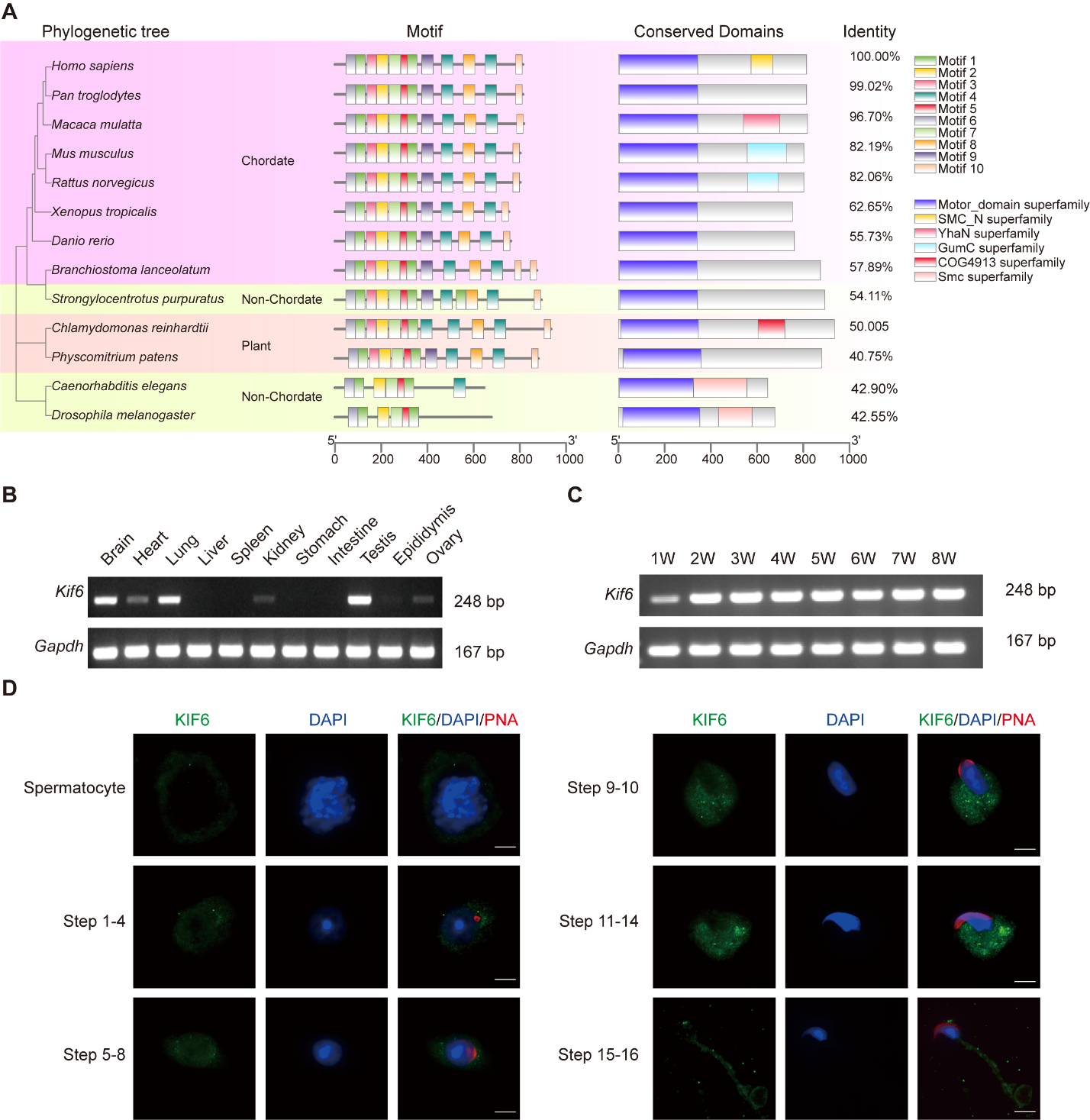


**Figure S2. Evolutionary conservation and expression patterns of the *Kif6* gene.** (**A**) Comparative sequence analysis of KIF6 across representative eukaryotic species spanning flagellated lineages, including lower plants and mammals. Species included in the analysis are indicated. (**B**) RT-PCR analysis of *Kif6* expression across adult mouse tissues, including testes and selected somatic tissues. (**C**) Developmental expression profile of *Kif6* in postnatal mouse testes measured by qRT-PCR. (**D**) Representative immunofluorescence images of KIF6 staining in selected spermatogenic cell types, including spermatocytes and elongating spermatids. Sections were prepared by the testicular squash method. Scale bars, 5 μm.

**
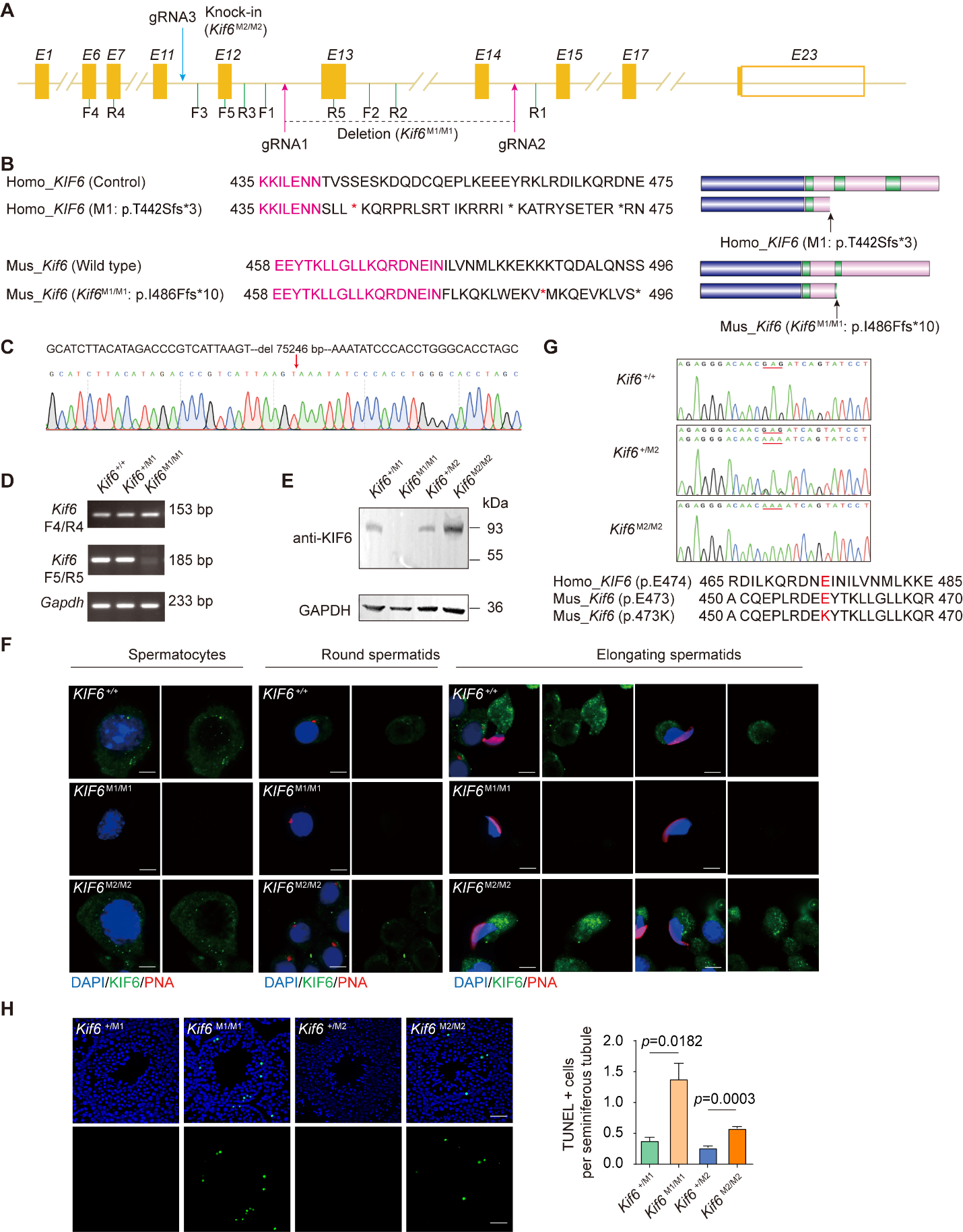
**

**Figure S3. Generation and validation of *Kif6* mutant mouse models (*Kif6*^M1/M1^ and *Kif6*^M2/M2^).** (**A**) Schematic overview of the CRISPR–Cas9 genome-editing strategy used to generate Kif6^M1/M1^ (frameshift) and Kif6^M2/M2^ (missense) mouse lines. The targeted genomic loci (exonic and intronic regions) are shown along with guide RNA (gRNA) sequences (purple for Kif6^M1/M1^, blue for Kif6^M2/M2^) and flanking PCR primers (forward [F], reverse [R]) used for genotyping. Genotyping was performed by PCR amplification followed by Sanger sequencing. (**B**) Schematic comparison of human KIF6 (NP_659464.3) and mouse KIF6 (NP_796026.2) protein domain architectures, indicating the truncating variant identified in individuals (p.T442Sfs*3) and the corresponding engineered mouse variant (p.I477Ffs*10). Red asterisks denote the positions of premature stop codons. (**C**) Representative Sanger sequencing chromatogram of *Kif6* genomic DNA from *Kif6*^M1/M1^ mice, showing the edited allele. The modified nucleotide position is indicated by a red arrow. (**D**) RT-PCR analysis of *Kif6* transcripts in testes from *Kif6*^+/+^, *Kif6*^+/M1^, and *Kif6*^M1/M1^ mice, using primers indicated in (A). n = 3 mice per genotype. € Representative Western blot analysis of KIF6 in testes from *Kif6*^+/M1^, *Kif6*^M1/M1^, *Kif6*^+/M2^, and *Kif6*^M2/M2^ mice. GAPDH served as a loading control. (**F**) Representative immunofluorescence staining of KIF6 in spermatogenic cells, including spermatocytes, round spermatids, and elongating spermatids, from *Kif6*^+/+^, *Kif6*^+/M1^, and *Kif6*^M1/M1^ mice. Sections were prepared by the testicular squash method. Scale bars, 5 µm. (**G**) Representative Sanger sequencing chromatogram of *Kif6* cDNA from *Kif6*^+/+^, *Kif6*^+/M2^, and *Kif6*^M2/M2^ mice, showing the nucleotide substitution corresponding to the mouse E473K variant. (**H**) Representative TUNEL staining of seminiferous tubule sections to detect apoptotic germ cells (top), and quantification of TUNEL-positive cells per tubule (bottom). **Data are presented as mean ± SEM (n = 3 mice per genotype). Scale bars, 40 µm. *P* values were calculated by unpaired two-tailed Student’s t-test with Welch’s correction (H).**

**
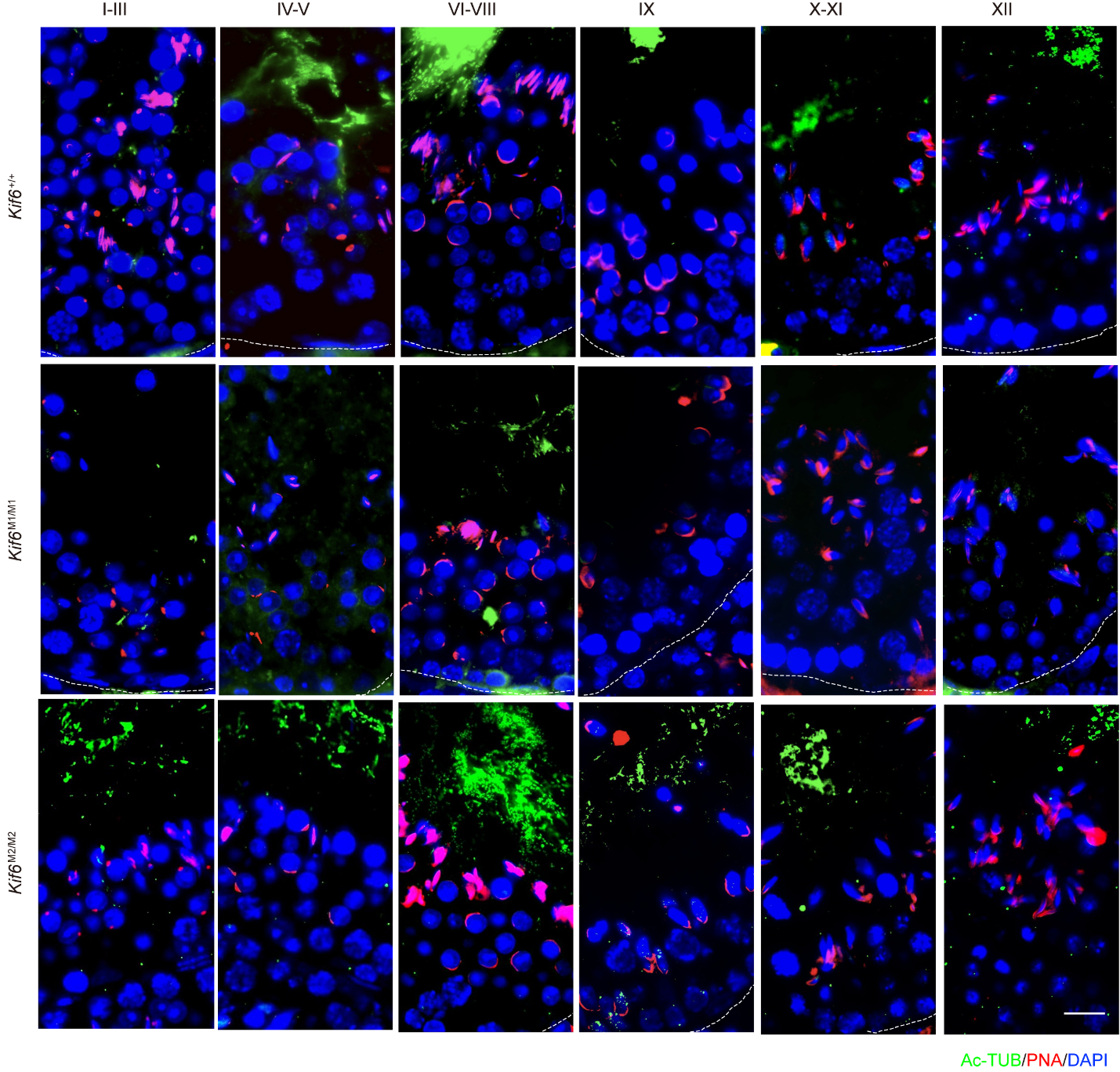
**

**Figure S4. PNA and acetylated tubulin co-staining reveals reduced flagellar signals in *Kif6* mutant seminiferous tubules.** Representative immunofluorescence images of seminiferous tubules at different developmental stages from *Kif6*^+/+^, *Kif6*^M1/M1^, and *Kif6*^M2/M2^ mice. Acetylated tubulin (Ac-TUB, green) marks flagellar structures, PNA (red) labels acrosomal structures, and DAPI (blue) labels nuclei. Scale bars, 20 μm.


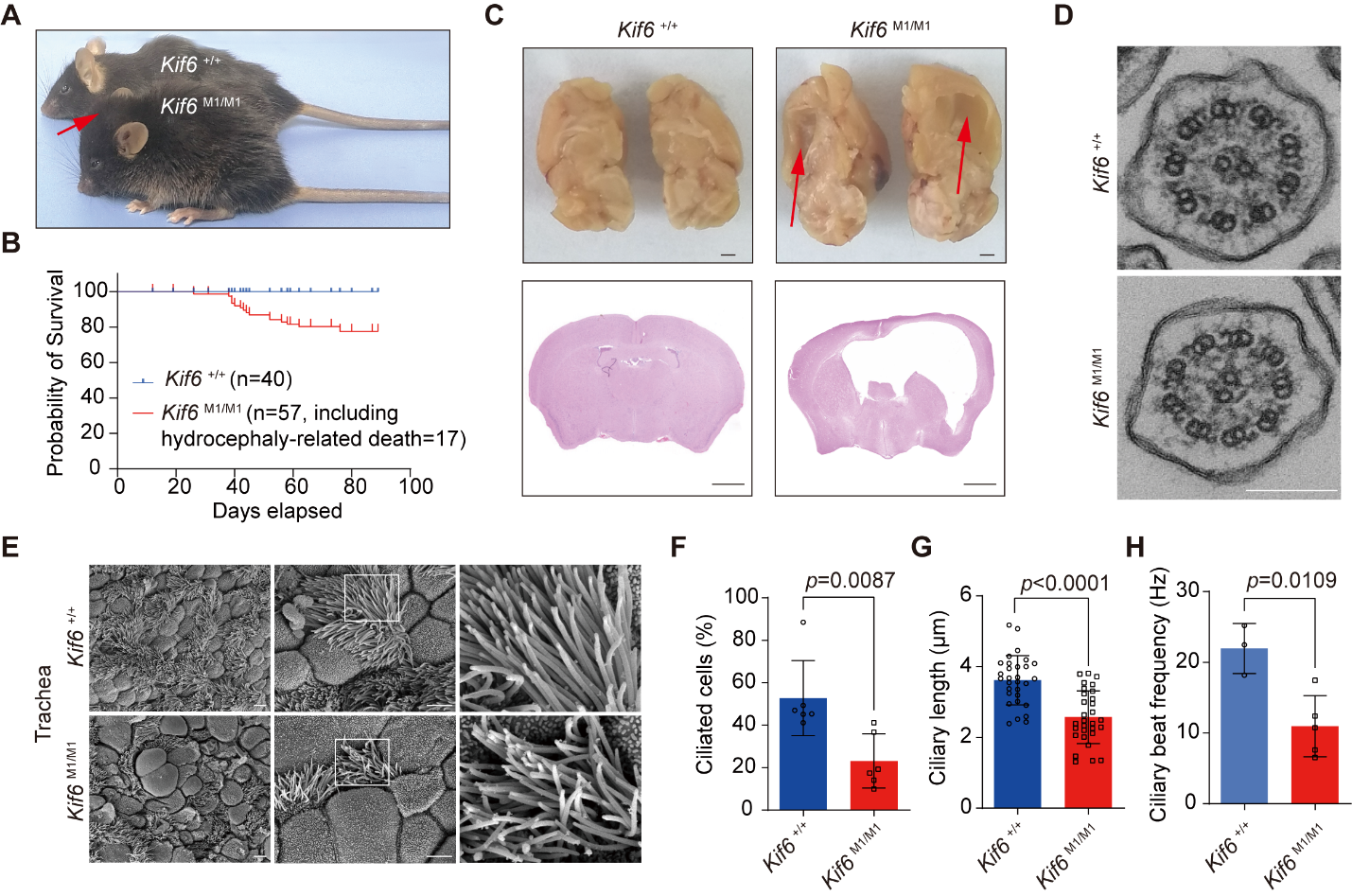


**Figure S5. Assessment of hydrocephalus and respiratory motile cilia in *Kif6*^M1/M1^ mice.** (**A**) Gross morphology of adult *Kif6*^+/+^ mice and hydrocephalic *Kif6*^M1/M1^ mice. (**B**) Survival analysis of *Kif6*^+/+^ mice and hydrocephalic *Kif6*^M1/M1^ mice. (**C**) Gross brain morphology and H&E staining of brain sections from *Kif6*^+/+^ mice and hydrocephalic *Kif6*^M1/M1^ mice. Scale bars, 1000 μm. (**D**) Transmission electron microscopy images of transverse sections of motile cilia in the tracheal epithelium. Scale bar, 100 nm. (**E**) Scanning electron microscopy images of tracheobronchial epithelial tissues from *Kif6*^+/+^ and hydrocephalic *Kif6*^M1/M1^ mice. Scale bars, 5 μm. (**F**, **G**) Quantification of the percentage of ciliated epithelial cells (**F**, n = 6 mice per group) and ciliary length (**G**, n = 30 cilia per group). (**H**) Quantification of ciliary beat frequency in tracheobronchial epithelial tissues from high-speed microscopy videos. *P* values were calculated by unpaired two-tailed Student’s t-test with Welch’s correction (F, G, H).


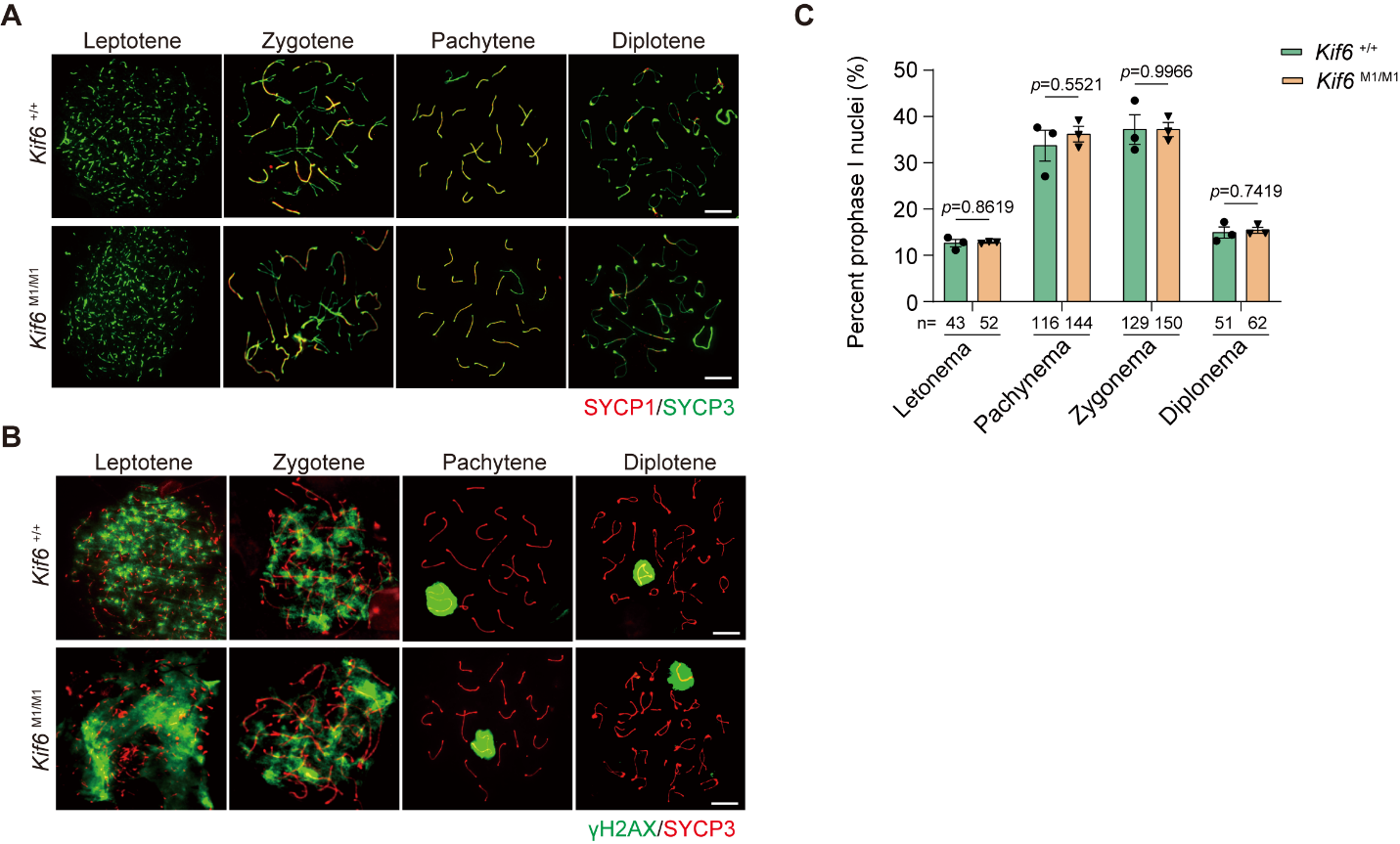


**Figure S6. Meiotic progression is unaltered in *Kif6*^M1/M1^ spermatocytes.** (**A**–**B**) Representative immunostaining images showing SYCP3 (A) and γH2AX (B) in meiotic chromosome from *Kif6*^+/+^ and *Kif6*^M1/M1^ spermatocytes. Scale bars, 20 μm. (**C**) Distribution of spermatocyte stages based on SYCP3 staining. A total of 339 and 408 spermatocytes were counted from *Kif6*^+/+^ and *Kif6*^M1/M1^ mice, respectively (n = 3 mice per genotype). *P* values were calculated by unpaired two-tailed Student’s t-test with Welch’s correction **(C)**.


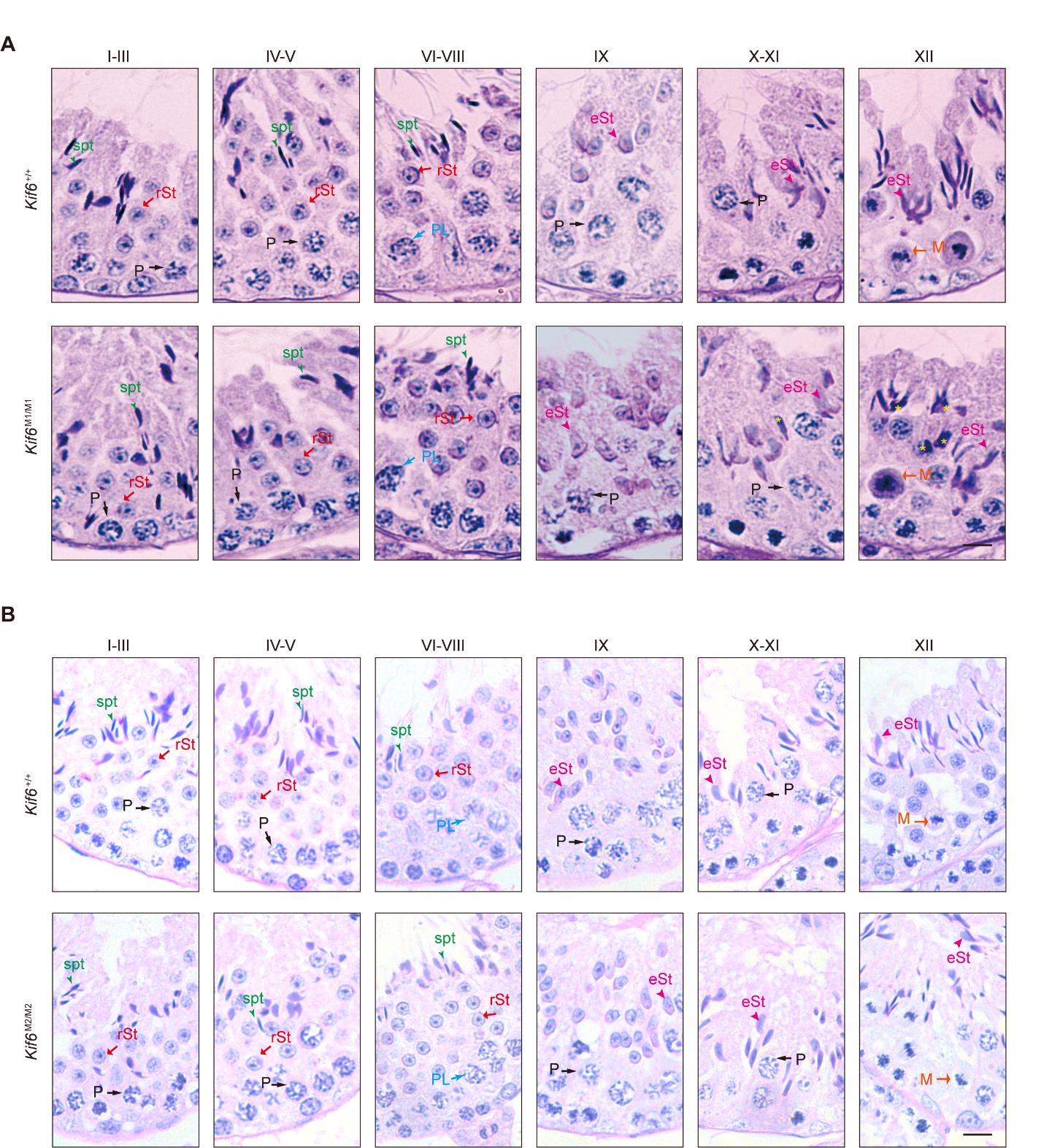


**Figure S7. PAS-based comparison of spermiogenesis in *Kif6*^M1/M1^ and *Kif6*^M2/M2^ mice.** (**A**) PAS-stained seminiferous tubule sections from adult *Kif6*^+/+^ and *Kif6*^M1/M1^ mice. Malformed elongating spermatids were observed in stage XI–XII seminiferous tubules of *Kif6*^M1/M1^ mice, as indicated by yellow asterisks. (**B**) PAS-stained testicular sections from 8-week-old *Kif6*^+/+^ and Kif6^M2/M2^ mice. Representative seminiferous tubule sections show largely preserved stage-associated spermatogenic morphology in Kif6^M2/M2^ mice. M, metaphase spermatocytes; P, pachytene spermatocytes; rSt, round spermatids; eSt, elongating spermatids. Scale bars, 10 µm.

**
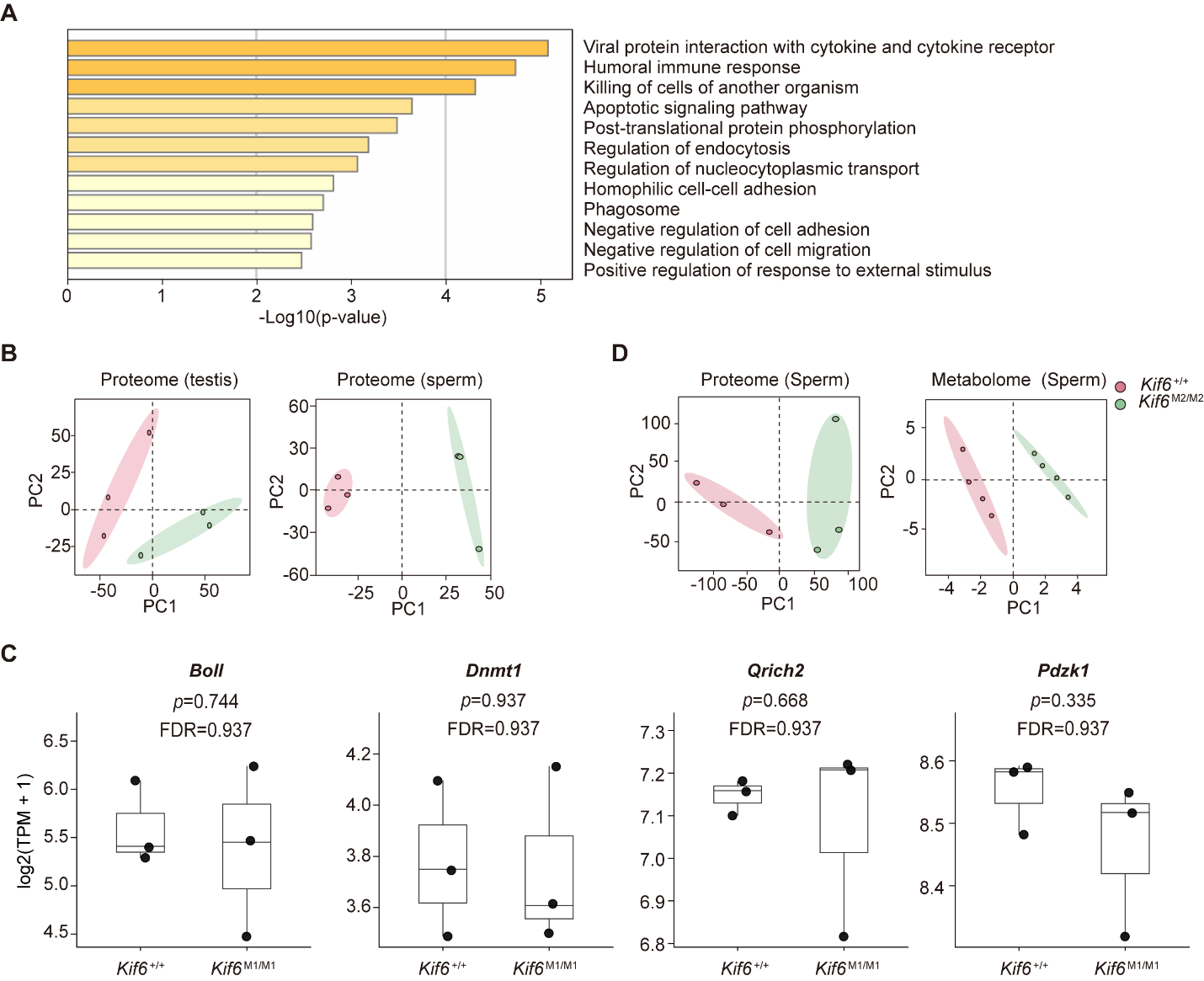
**

**Figure S8. Global transcriptomic, proteomic, and metabolomic profiling of *Kif6* mutant mice.** (**A**) Metascape functional enrichment analysis of DEGs in adult testes comparing Kif6^+/+^ and Kif6^M1/M1^ mice. (**B**) PCA mass-spectrometry-based proteomic data obtained from adult testes of Kif6^+/+^ and Kif6^M1/M1^ mice (n = 3 mice per genotype). Shaded ellipses represent the 95% confidence interval for each genotype. (**C**) Dot plots showing the RNA-seq expression levels of *Boll*, *Dnmt1*, *Qrich2*, and *Pdzk*1 in Kif6^+/+^ and *Kif6*^M1/M1^ testes. Each point represents one biological sample. Expression values are shown as log2(TPM + 1). P values were calculated using Welch’s t-test, and FDR values were adjusted using the Benjamini–Hochberg method. (**D**) PCA of proteomic (*n* = 3 mice per genotype) and metabolomic (*n* = 4 mice per genotype) datasets generated from caudal sperm of *Kif6*^+/+^ and *Kif6*^M2/M2^ mice.


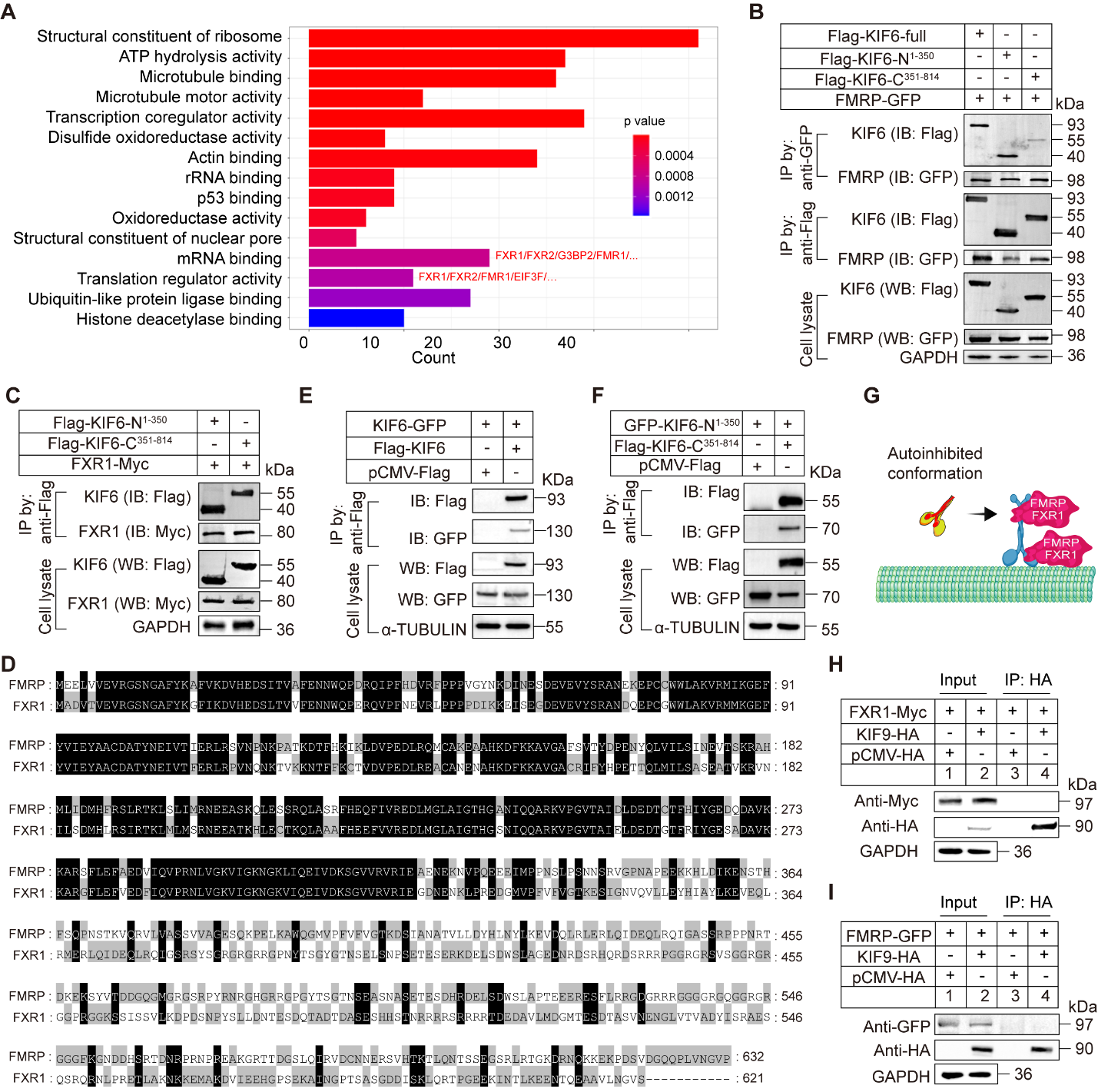


**Figure S9. Interaction mapping, domain analysis, and structural features of KIF6 and associated RNA-binding proteins.** (**A**) GO enrichment analysis of the 262 proteins identified as KIF6-associated by IP-MS. (**B–C**) Co-IP analysis of epitope-tagged KIF6 truncation constructs expressed in HEK293T cells, assessing their association with FMRP (B) or FXR1 (C). (**D**) Multiple sequence alignment of FMRP and FXR1 highlighting conserved regions within their N-terminal domains. (**E**) Co-IP analysis of differently tagged KIF6 constructs expressed in HEK293T cells, assessing association between KIF6 molecules. (**F**) In vitro interaction assays examining association between the N-terminal motor region and the C-terminal coiled-coil region of KIF6. (**G**) Schematic representation of a proposed KIF6 activation cycle, in which association with RNA-binding proteins is accompanied by changes in intramolecular configuration and microtubule association. (**H-I**) Co-IP analysis of epitope-tagged KIF9 and FMRP (H) or FXR1 (I) expressed in HEK293T cells.


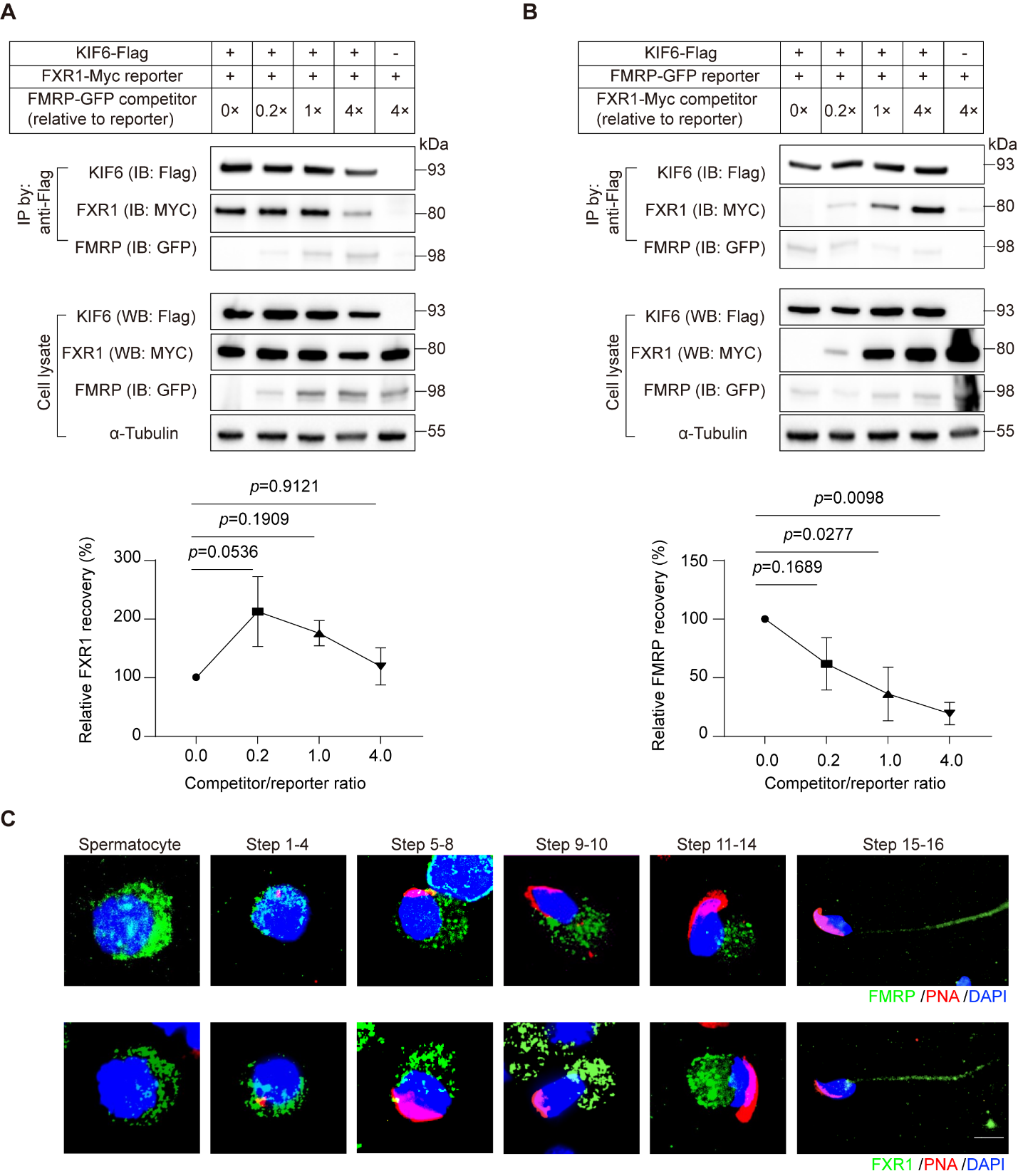


**Figure S10. FMRP and FXR1 show differential competitive association with KIF6 and distinct expression patterns during spermatogenesis.** (**A**) Relative recovery of FXR1 in KIF6 immunoprecipitates when increasing amounts of FMRP were introduced as a competitor. The competitor/reporter ratio is indicated on the x-axis. FXR1 recovery was calculated from the FXR1 signal in KIF6 immunoprecipitates after normalization to immunoprecipitated KIF6 and corresponding input FXR1 levels, and then expressed relative to the no-competitor condition, which was set as 100%. (**B**) Relative recovery of FMRP in KIF6 immunoprecipitates when increasing amounts of FXR1 were introduced as a competitor. FMRP recovery was calculated from the FMRP signal in KIF6 immunoprecipitates after normalization to immunoprecipitated KIF6 and corresponding input FMRP levels, and then expressed relative to the no-competitor condition, which was set as 100%. (**C**) Representative immunofluorescence images showing the expression patterns of FMRP and FXR1 during spermatogenesis. PNA staining was used to mark developing spermatids. Scale bars, 5 μm. For A and B, data are shown as mean ± SEM. *P* values were calculated by one-way ANOVA with Dunnett’s multiple comparisons test (A, B).


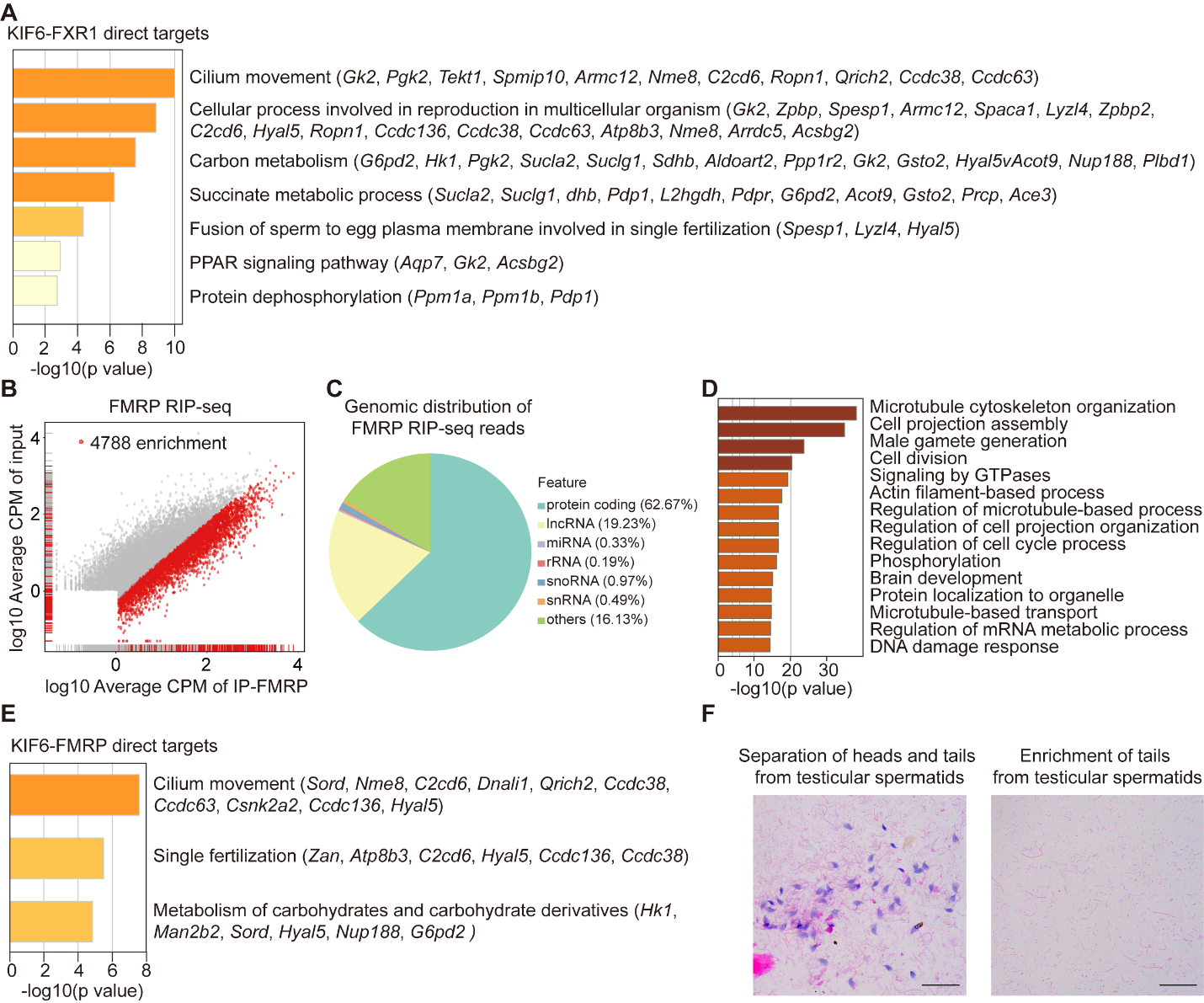


**Figure S11. Functional characterization of KIF6–FMRP/FXR1 mRNA cargoes.** (**A**) GO enrichment analysis of the 59 transcripts identified from the intersection between FXR1-associated transcripts and proteins showing reduced abundance in sperm from *Kif6* mutant mice. (**B**) Scatter plot of RIP-seq performed using anti-FMRP antibodies on the adult WT mice testes, showing enrichment of immunoprecipitated RNA relative to input RNA. Transcripts with fold change > 1.5 and false discovery rate (FDR) < 1% are highlighted in red. CPM, counts per million. (**C**) Pie charts showing the genomic distribution of FMRP RIP-seq reads across RNA classes. (**D**) Metascape gene annotation analysis of transcripts identified as FMRP-associated in the RIP–seq dataset. (**E**) GO enrichment analysis of the 26 transcripts identified from the intersection between FMRP-associated transcripts and proteins showing reduced abundance in sperm from *Kif6* mutant mice. (**F**) Representative H&E-stained images of isolated flagellar fractions from testicular spermatids. Scale bars, 20 μm.


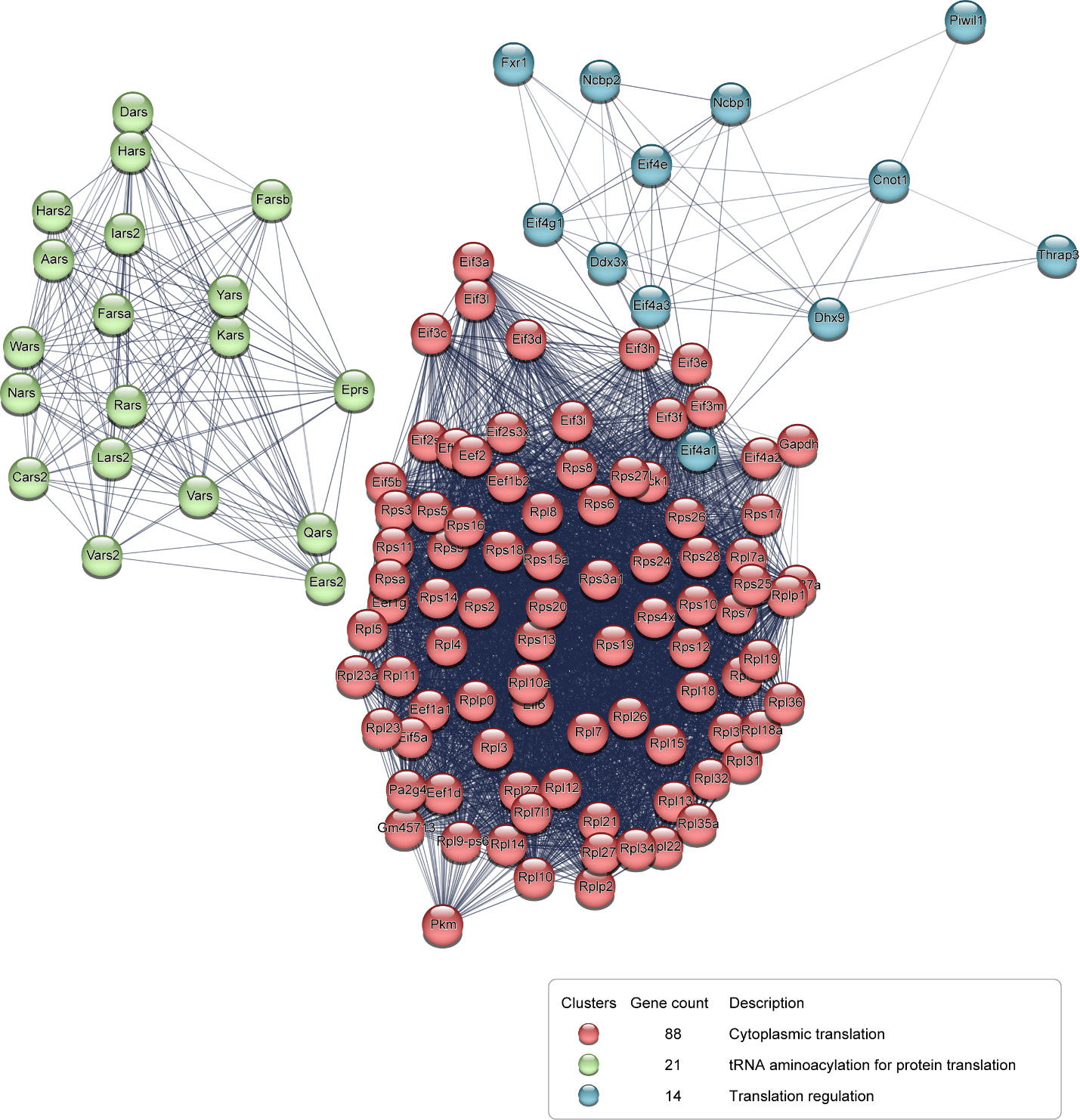


**Figure S12. Protein–protein interaction network analysis of cytoplasmic translation-related components identified in sperm flagella.** Cytoplasmic translation-related components detected in the flagellar proteome were analyzed for protein–protein interaction (PPI) and network clustering using STRING, with clustering performed using the legacy k-means algorithm. Distinct clusters related to cytoplasmic translation, tRNA aminoacylation for protein translation, and translation regulation were identified, indicating non-random associations and coordinated organization among translation-related components in sperm flagella.


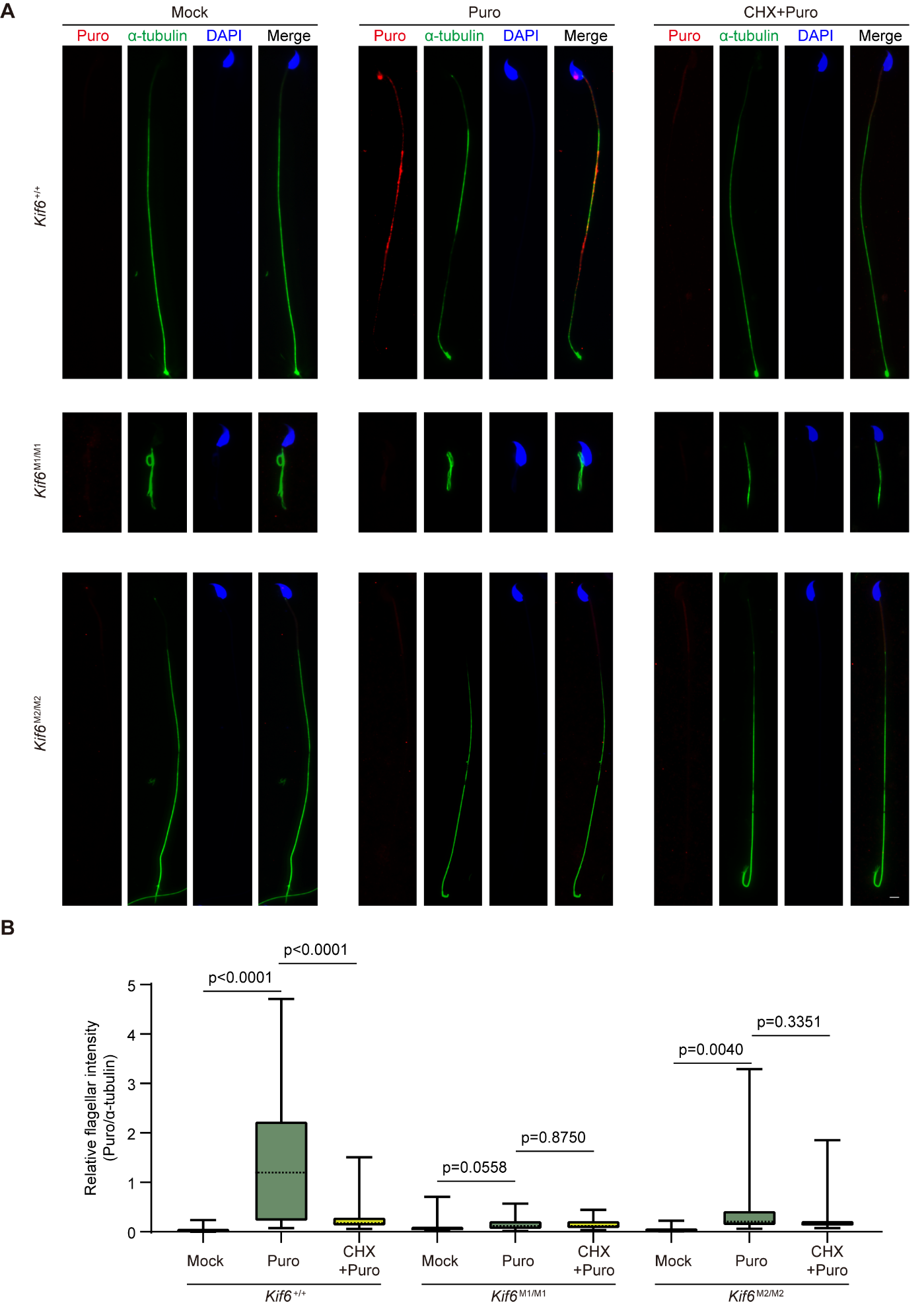


**Figure S13. Puromycin labeling reveals reduced nascent peptide synthesis in *Kif6* mutant sperm flagella.** (**A**) Representative immunofluorescence images of live epididymal sperm from WT, *Kif6*^M1/M1^, and *Kif6*^M2/M2^ mice after mock treatment, puromycin treatment, or combined cycloheximide (CHX) and puromycin treatment. Puromycylated nascent peptides were detected using an anti-puromycin antibody. α-tubulin was used as a flagellar marker, and DAPI labeled sperm DNA. Scale bar, 5 μm. (**B**) Quantification of mean flagellar anti-puromycin fluorescence intensity in the indicated groups. n = 30 sperm per group. ***P* values were calculated by unpaired two-tailed Student’s t-test with Welch’s correction (B).**


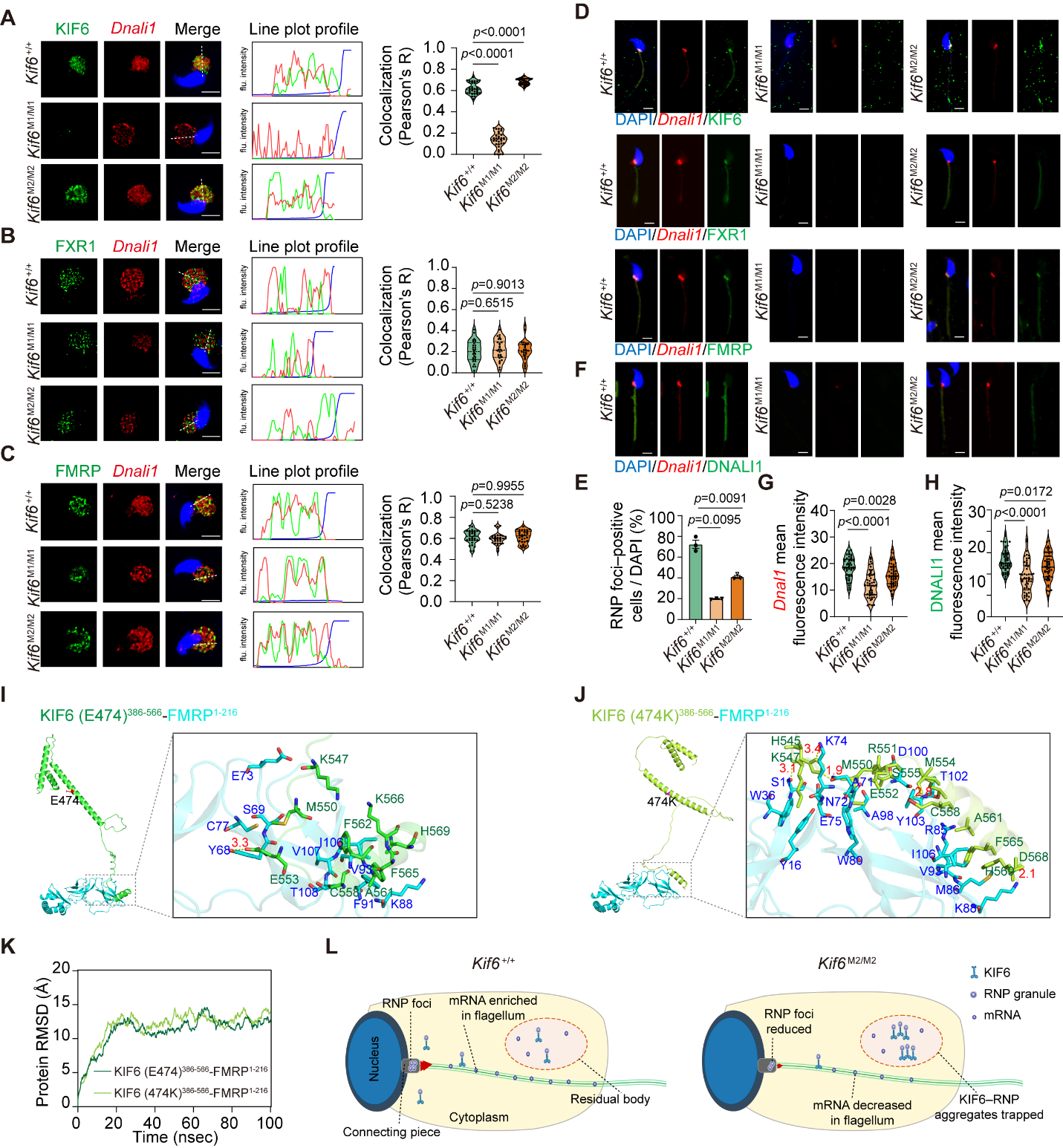


**Figure S14.** ***Kif6* mutations reduce RBP-dependent cargo mRNA delivery to the flagellum via the connecting piece and disrupt flagellar protein expression.** (**A–C)** Immuno-FISH of KIF6 (A), FXR1 (B) or FMRP (C) proteins (green) with Dnali1 mRNA (red) in isolated elongating spermatids from *Kif6*^+/+^, *Kif6*^M1/M1^, and *Kif6*^M2/M2^ mice. Representative images (left), line-scan intensity profiles (middle) and co-localization quantification (right; n = 30 spermatids per genotype). (**D)** Representative immuno-FISH showing subcellular distribution of Dnali1 RNP signals. (**E)** Percentage of spermatids with Dnali1 RNP signals at the connecting piece (mean ± SEM; n = 3 mice per genotype; 100 spermatids per mouse). (**F)** Immuno-FISH of Dnali1 mRNA (red) and DNALI1 protein (green) in spermatids of the indicated genotypes. (**G,H)** Quantification of Dnali1 mRNA (G) and DNALI1 protein (H) fluorescence intensity along the flagellar compartment in elongating spermatids (mean ± SEM; n = 50 spermatids per genotype). (**I,J)** Molecular docking models of the KIF6–FMRP interaction interface for WT (I) and mutant (J) complexes, highlighting residues contributing to the predicted interaction surface; WT binding included a hydrogen bond between KIF6-E553 and FMRP-Y68, whereas the mutant interface gained additional predicted contacts. (**K)** RMSD analysis of docked KIF6–FMRP complexes. (**L)** Model illustrating the impact of the *Kif6* missense mutation on mRNA trafficking: in WT spermatids, KIF6–mRNP granules accumulate at the connecting piece and are delivered into the flagellum, whereas strengthened KIF6–RBP association in the mutant promotes cytoplasmic retention and reduces flagellar mRNA delivery. Scale bars, 5 μm (A–F). In A–C, each dot represents the percentage of protein signal co-localizing with mRNA signal in an individual spermatid; in G,H, each dot represents the mean flagellar fluorescence intensity from a single spermatid. A–C, G–H, n = 3 biologically independent experiments. *P* values were calculated by one-way ANOVA with Dunnett T3 correction for multiple comparisons (A–C, E, G, H).

Table S1. Kinesin family variants identified in WES screening of men with asthenozoospermia.

| Kinesin Family Candidate Gene | Clinical Mutation Characteristics & Expression/Function Background |
| --- | --- |
| *KIF6*  exon12:c.1325_1326del:p.T442Sfs*4  exon12:c.1420G>A:p.E474K | Biallelic variants identified in two affected individuals. Highly expressed in human testes. Localizes to axonemal microtubules of motile cilia and exhibits microtubule-based motility. *Kif6* knockout mice show normal sperm morphology but severely impaired motility (67% immotile vs. 3% in WT);^[34]^ however, the underlying mechanism remains unknown. |
| *KIF23*  exon19:c.2296G>T:p.E766X  exon19:c.2296G>T:p.E766X | According to the Human Protein Atlas, KIF23 is widely expressed across tissues, including testes. |
| *KIF20B*  exon8:c.901C>T:p.R301C | Biallelic variants identified in one affected individuals.Highly expressed in testis, but no studies have linked it to sperm function or motility; its relevance to male infertility remains unknown. |
| *KIF13B*  exon8:c.706G>T:p.D236Y | Human Protein Atlas data show no detectable expression of KIF13B in human spermatogenic cells. |

Table S2. Semen routine parameters and sperm flagellar morphology in men harboring homozygous deleterious *KIF6* variants.

| **Semen parameter** | **T00521 Ⅱ-1** | **T01046 Ⅱ-1** | **Reference Limits** ^[63,72]^ |
| --- | --- | --- | --- |
| Semen volume (mL) | 1.8 | 2.5 | >1.5 |
| Sperm concentration (10^6^/mL) | 1.27 | 23.44 | >15 |
| Motility (%) | 0 | 0 | >40 |
| Progressive motility (%) | 0 | 0 | >32 |
| **Sperm morphology** |  |  |  |
| Normal spermatozoa (%) | 1.8 | 3.03 | >4 |
| Absent flagella (%) | 16.39 | 7.88 | ≤5 |
| Short flagella (%) | 19.67 | 12.33 | ≤1 |
| Coiled flagella (%) | 40.16 | 27.39 | ≤17 |
| Angulation (%) | 13.11 | 3.42 | ≤13 |
| Irregular caliber (%) | 4.09 | 9.59 | ≤2 |

**Table S3.** **Bi-allelic *KIF6* variants identiﬁed in chinese men with asthenozoospermia.**

|  | Subject |  |
| --- | --- | --- |
|  | T00521 Ⅱ-1 | T01046 Ⅱ-1 |
| Gene | KIF6 | KIF6 |
| cDNA alteration | c.1325_1326del | c.1420G>A |
| Protein alteration | p.Thr442Serfs*3 | p.Glu474Lys |
| Variant type | frameshift deletion | nonsynonymous SNV |
| **Allele frequency in human populations** |  |  |
| ExAC | 0 | 0.0002 |
| 1000 Genomes Project | 0 | 0.000199680999999999 |
| GnomAD_exome (All) | 0 | 0.0002 |
| GnomAD_exome (East Asian) | 0 | 0.0039 |
| 1,500 fertile controls | 0 | 0 |
| **Conservation of the affected amino acid residues** |  |  |
| PhyloP | N/A | 4.335 |
| PhastCons | N/A | 1 |
| **Functional prediction** |  |  |
| SIFT | N/A | D |
| PolyPhen-2 | N/A | D |
| Mutation Taster | N/A | D |

KIF6, kinesin family member 6; ExAC, Exome Aggregation Consortium; SIFT, Sorting Intolerant from Tolerant; N/A, not applicable.

**Table S4. Clinical outcomes of ICSI cycles using the spermatozoa from men harboring homozygous deleterious *KIF6* variants.**

| **Subject** | **T00521 Ⅱ-1** | **T01046 Ⅱ-1** |
| --- | --- | --- |
| Male age (year) | 29 | 41 |
| Female age (year) | 22 | 30 |
| Number of ICSI cycles | 1 | Donor |
| Number of oocytes injected | 12 |  |
| Number (and rate) of fertilized oocytes | 8(67%) |  |
| Number (and rate) of cleavage embryos | 8(100%) |  |
| Number (and rate) of 8-cells | 6(75%) |  |
| Number (and rate) of blastocysts | 6(75%) |  |
| Number of transfer cycles | 1 |  |
| Number of embryos transferred per cycle | 2 |  |
| Implantation rate | 50% |  |
| Clinical pregnancy rate | 100% |  |
| Miscarriage rate | NA |  |

NA, not available.

**Table S5. Primers used for the amplification and verification of *KIF6* variants.**

| **Primer Name** | **Forward primer (5'-3')** | **Reverse primer (5'-3')** |
| --- | --- | --- |
| *KIF6*-M1 | GTGTGCAGGGAATTGTTAAAGGA | TGCTGAAGGGACCTTTGACTTC |
| *KIF6*-M2 | ACTGCATCGCAAAGACATCG | TTTGCTGAAGGGACCTTTGACT |

**Table S6. Primers used for mouse *Kif6* genotyping.**

| **Primer Name** | **Primer Sequence (5'-3')** | **Reverse primer (5'-3')** |
| --- | --- | --- |
| *Kif6*-M1-1 (F1/R1) | TGTTCTGAGTAGTAGCCATCTCT | TGTGCAAGCATCTAATCCATCAC |
| *Kif6*-M1-2 (F2/R2) | CTAAGGCATTTAGCCTGGGAGAA | GGTACCCTCGCTTGCTTACTC |
| *Kif6*-M2-3 (F3/R3) | AGTGTGTACTAGGAACAGCAGAAA | TCCTTTGTGAGGATAACTGTGTCA |

**Table S7. Primers used for RT-PCR analysis.**

| **Gene** | **Primer Sequence (5'-3')** | **Reverse primer (5'-3')** |
| --- | --- | --- |
| *Kif6*-4 (F4/R4) | TGGAGGACCCTGATCAGAAC | TACAGTGTGACCGGGTTGAA |
| *Kif6*-5 (F5/R5) | CATGCCAAGAACCATTGAGA | GATGAAGGGCGAGTTCTGAG |
| *Gapdh* | TCATCATCTCCGCCCCTTCT | CTGGGTGGCAGTGATGGCAT |
| *Boll* | CATCTTGGCCTTCACGTTCC | TCAGAGACAGTGGAGGAGGA |
| *Dnmt1* | CGGCTCAAAGACTTGGAAAG | TAGCCAGGTAGCCTTCCTCA |
| *Pdzk1* | ATGAAAGCTGGTGTTCTGGC | CTGGCTGTCTCCCTCTTGAA |
| *Qrich2* | AAAGACCATACCCCAGGACC | ACACGGTGACTCTGAGGATG |
| *Sord* | TGCGGCTCAGATGTTCACTA | TACCGGCCAATCTTGCAGTA |
| *Nme8* | CTTCACTTCGTCGTTGCTGA | GACGATCCATTTCACCTGCT |
| *Dnali1* | TAAATGCCATCCTGCCTCCA | TCCCGGATCAGTTCGTCAAA |
| *Hk1* | TGCCATGCGGCTCTCTGATG | CTTGACGGAGGCCGTTGGGTT |

**Table S8. Antibodies used for this study.**

| **Antibody** | **Source** | **Identifier** |
| --- | --- | --- |
| DDDDK-Tag Rabbit mAb | ABclonal | Cat# AE092, RRID:AB_2940847 |
| Flag-Tag Mouse Monoclonal Antibody | Abways Technology | Cat# AB0008, RRID:AB_2943672 |
| α-Tubulin Mouse mAb | ABclonal | Cat# AC012 |
| KIF6 Polyclonal antibody | Proteintech | Cat# 17290-1-AP |
| BOLL Polyclonal Antibody | ABclonal | Cat# A8255, RRID:AB_2768611 |
| GAPDH antibody | Abcam | Cat# ab8245, RRID:AB_2107448 |
| Monoamine Oxidase B Rabbit mAb (anti-MAOB) | ABclonal | Cat# A11597, RRID:AB_2861605 |
| EIF3K Polyclonal Antibody | ABclonal | Cat# A9969, RRID:AB_2769293 |
| CYC1 Polyclonal Antibody | ABclonal | Cat# A10449, RRID:AB_2757996 |
| PPP1CA Polyclonal Antibody | ABclonal | Cat# A12468, RRID:AB_2759312 |
| PGK2 Polyclonal Antibody | ABclonal | Cat# A12952, RRID:AB_2759798 |
| NDUFA12 Polyclonal Antibody | ABclonal | Cat# A8237, RRID:AB_2770544 |
| DYNLL1 Rabbit mAb | ABclonal | Cat# A4353, RRID:AB_2863246 |
| DYNLL2 Polyclonal Antibody | ABclonal | Cat# A13888, RRID:AB_2760741 |
| HK1 Rabbit mAb | ABclonal | Cat# A0533, RRID:AB_2861463 |
| Anti-Cenexin1/ODF2 Antibody | Abcam | Cat# ab43840 |
| NDUFA8 Polyclonal Antibody | ABclonal | Cat# A12118, RRID:AB_2759008 |
| Lamin A/C Polyclonal Antibody | ABclonal | Cat# A0249, RRID:AB_2757062 |
| FMRP antibody | Abcam | Cat# ab17722, RRID:AB_2278530 |
| FXR1 Antibody | Novus | Cat# NBP2-22246, RRID:AB_3068565 |
| FXR2 Polyclonal Antibody | ABclonal | Cat# A4313, RRID:AB_2765617 |
| GFP tag Ab | Abways | Cat# AB0005 |
| Myc tag Ab | Abways | Cat# AB0001 |
| QRIC2 Rabbit pAb (Anti-QRICH2) | Immnuoway | Cat# YN5003 |
| Sorbitol Dehydrogenase Rabbit mAb (anti-SORD) | ABclonal | Cat# A21952, RRID:AB_2936952 |
| NME8 Polyclonal Antibody | ABclonal | Cat# A17153, RRID:AB_2770603 |
| Goat Anti-Rabbit IgG (H+L) HRP | Affinity Biosciences | Cat# S0001, RRID:AB_2839429 |
| Goat Anti-Mouse IgG (H+L) HRP | Affinity Biosciences | Cat# S0002, RRID:AB_2839430 |
| Anti-DNALI1 Antibody | Abcam | Cat# ab155490 |
| HA tag Ab | Abways | Cat# AB0004 |
| Goat anti-Rabbit IgG (H+L) Highly Cross-Adsorbed Secondary Antibody, Alexa Fluor™ 555 | Thermo Fisher Scientific | Cat# A-21429, RRID:AB_2535850 |
| Goat anti-Mouse IgG (H+L) Highly Cross-Adsorbed Secondary Antibody, Alexa Fluor™ 488 | Thermo Fisher Scientific | Cat# A-11029, RRID:AB_2534088 |
| Rabbit IgG | Beyotime | Cat# A7016, RRID:AB_2905533 |
| KIF6 Polyclonal Antibody | Thermo Fisher Scientific | Cat# PA5-66190, RRID:AB_2663765 |
| Rabbit Anti-SCP1 Polyclonal Antibody | Abacm | Cat# ab15090, RRID:AB_301636 |
| Mouse Monoclonal SCP3 antibody | Abcam | Cat# ab205846 |
| gamma H2A.X (phospho S139) antibody | Abcam | Cat# ab11174, RRID:AB_297813 |
